# Dietary Alteration of the Gut Microbiome and Its Impact on Weight and Fat Mass: A Systematic Review and Meta-Analysis

**DOI:** 10.3390/genes9030167

**Published:** 2018-03-16

**Authors:** George Kunnackal John, Lin Wang, Julie Nanavati, Claire Twose, Rajdeep Singh, Gerard Mullin

**Affiliations:** 1 Johns Hopkins University School of Medicine, Baltimore, MD 21205, USA; gjohn3@jhmi.edu (G.K.J.); jnanava1@jhmi.edu (J.N.); ctwose@jhmi.edu (C.T.); 2 Johns Hopkins School of Public Health, Baltimore, MD 21205, USA; linwang@jhu.edu; 3 Sinai Hospital, Baltimore, MD 21215, USA; rajdeepw17@gmail.com

**Keywords:** gut microbiome, probiotics, weight loss, obesity treatment

## Abstract

Dietary alteration of the gut microbiome is an important target in the treatment of obesity. Animal and human studies have shown bidirectional weight modulation based on the probiotic formulation used. In this study, we systematically reviewed the literature and performed a meta-analysis to assess the impact of prebiotics, probiotics and synbiotics on body weight, body mass index (BMI) and fat mass in adult human subjects. We searched Medline (PubMed), Embase, the Cochrane Library and the Web of Science to identify 4721 articles, of which 41 were subjected to full-text screening, yielding 21 included studies with 33 study arms. Probiotic use was associated with significant decreases in BMI, weight and fat mass. Studies of subjects consuming prebiotics demonstrated a significant reduction in body weight, whereas synbiotics did not show an effect. Overall, when the utilization of gut microbiome-modulating dietary agents (prebiotic/probiotic/synbiotic) was compared to placebo, there were significant decreases in BMI, weight and fat mass. In summary, dietary agents for the modulation of the gut microbiome are essential tools in the treatment of obesity and can lead to significant decreases in BMI, weight and fat mass. Further studies are needed to identify the ideal dose and duration of supplementation and to assess the durability of this effect.

## 1. Introduction

Current data estimate that approximately 600 million people around the world are obese, with an additional 1.9 billion overweight people [1]. Obesity is a significant risk factor for several health conditions and is a major cause of mortality and morbidity across the globe. Thus, obesity can lead to cardiovascular disease, hypertension, diabetes and various cancers [2,3]. The development of obesity is multifactorial and is influenced by genetic, individual and environmental risk factors [4]. As recently discovered, one of the most important risk factors affecting obesity is the influence of the gut microbiome [5,6]. The human gut microbiome consists of several trillion microbes, which reside in the gastrointestinal tract with their genes that code for a wide array of effects on human physiology [5]. Gut microbes ferment non-digestible polysaccharides, thereby producing short-chain fatty acids (SCFAs), which bind to the GPR 41/43 receptors on gut epithelial cells and stimulate peptide YY (PYY) and glucagon-like peptide-1 (GLP-1) production [6]. PYY and GLP-1 are gut-derived hormones that attenuate gut motility and facilitate the aggregation of the constitutive flora to ferment more polysaccharides. These gut-hormones also suppress appetite by delaying gastric emptying and centrally promoting satiation [7]. SCFAs also promote gut barrier integrity and antagonize local and systemic inflammation, which drives insulin resistance and lipogenesis [8,9]. Gut microbiota regulate energy metabolism by downregulating the expression of fasting-induced adipocyte factor (Fiaf) from gut epithelial cells, thus resulting in the degradation of lipoproteins and the deposition of free fatty acids in adipose tissues [10]. The adiposity in the liver and skeletal muscles is also regulated by gut microorganisms via phosphorylated adenosine monophosphate-activated protein kinase (AMPK) levels [6]. In health, constitutive gut microbes maintain immunoglobulin A (IgA) levels to prevent colonization by enteric pathogens [11]. Finally, the protective mucin layer is enhanced by the gut microbe *Akkermansia muciniphila*, which appears to protect the host against the invasion of pathogens, the breakdown of gut barrier defenses, systemic inflammation, endotoxemia, atherogenesis, lipogenesis and insulin resistance and its consequences [12,13]. The gut microbiome has a crucial role in the functioning of the digestive tract and in harvesting energy from the diet [14,15]. There is a large body of evidence demonstrating the link between the gut microbiome and obesity, with one of the most frequently-cited contributing factors being a shift in the proportion of bacterial flora belonging to the *Firmicutes* relative to *Bacteroidetes* phyla [16,17]. Bacteria belonging to the *Firmicutes* phyla are known to be more efficient extractors of highly caloric short-chain fatty acids from fiber relative to *Bacteroidetes* [18]. Previous animal and human studies have shown a correlation between the increasing proportion of stool *Bacteroidetes* with weight loss and *Firmicutes* with the development of obesity [17,19,20,21]. Furthermore, fecal transplantation experiments in animals were able to successfully confer obesity by transplanting the stool from leptin deficient (ob/ob) mice harboring higher concentrations of the *Firmicutes* phyla of gut microbes [16]. 

**Figure 1:**
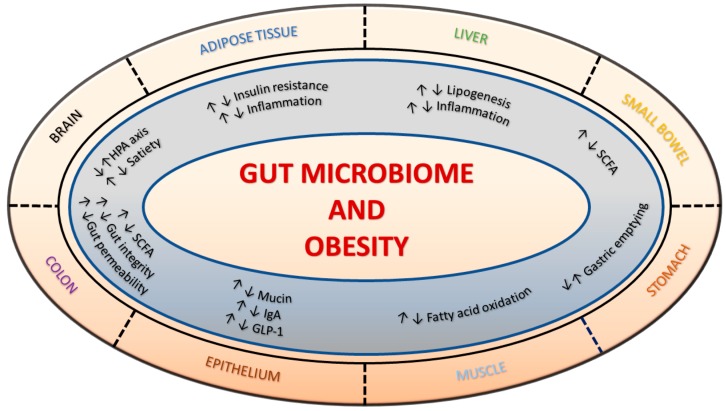
Influence of the gut microbiome on obesity. The figure depicts the bidirectional influence of gut health on obesity via alterations in the microbiome. SCFA: short-chain fatty acid; IgA: immunoglobulin A; GLP-1: glucagon-like peptide-1; HPA: hypothalamic–pituitary–adrenal (axis).

The ability to engineer a favorable metabolic environment by dietary modulation makes the gut microbiome an attractive target in the war against obesity (Figure 1) [22,23,24,25]. Dietary modulation of the gut microbiome includes three kinds of foods: prebiotics, probiotics and synbiotics [26]. Prebiotics are nonviable food components associated with the favorable modulation of the gut microbiota, such as inulin, fructo-oligosaccharides, galacto-oligosaccharides, resistant starch, xylo-oligosaccharides and arabinoxylan-oligosaccharides [27]. Probiotics are living microorganisms, such as *Lactobacillus* and *Bifidobacterium*, which, when ingested, provide health benefits, either directly or through interactions with the host or other microorganisms [28]. The combination of pre- and pro-biotics has been termed synbiotics. Previous randomized controlled trials using probiotics have demonstrated varying strain-specific effects on body weight and body mass index (BMI) [29]. The use of prebiotics has been demonstrated to decrease post-prandial glucose and insulin, but its effect on body weight has been contradictory in different studies [30]. 

In this study, we aimed to review the literature systematically for evidence from randomized controlled trials on the impact of prebiotics, probiotics and synbiotics on weight, BMI and fat mass in adult human subjects and to analyze the overall effect based on pooled data from these trials. 

## 2. Methods

### 2.1. Protocol and Registration

This study was registered in the International Prospective Register of Systematic Reviews (PROSPERO 2017 CRD42017075883) and the protocol is available for download at [31].

### 2.2. Search Strategy

The search strategy was based on input from the authors, key articles and the pearl growing of terms based on preliminary search results. There were no restrictions placed on publication dates or language. All searches were executed on 16 August 2017 from the following databases: Medline (PubMed) [32], Embase [33], the Cochrane Library (Cochrane Database of Systematic Reviews, Cochrane Central Register of Controlled Trials (CENTRAL) [31], Cochrane Methodology Register and the Web of Science (Science and Social Science Citation Index) [34]. For the search strategies designed for Medline (PubMed), the Cochrane Library and Embase, the controlled vocabulary terms for each concept were identified and combined with keyword synonyms. The Web of Science was searched using keyword terms only (see Table 1 for exact search strategies). Pertinent searches of references of review papers and grey literature were also conducted, and 20 additional records were identified from this process. 

### 2.3. Eligibility Criteria

All interventional trials (randomized or non-randomized) utilizing prebiotics/probiotics/synbiotics in supplement or food-based form in adult human individuals (18 and older) with BMI ≥ 25 (overweight and obese) were included, while studies with children or pregnant patients were excluded. The use of any food or supplement influencing the gut microbiome (probiotic, prebiotic, synbiotic) was considered as the exposure, and weight, BMI and fat mass changes in individuals following dietary intervention were the primary outcomes.

### 2.4. Data Extraction 

The titles and abstracts of the studies were reviewed independently by two individual reviewers based on the inclusion and exclusion criteria. Consensus resolved any disagreement(s) when needed by input from a third reviewer. Articles deemed to have met the aforementioned criteria were then subjected to a full-text review. Data extraction was completed by two extractors. A third author served as a referee for resolving the disputes and cross-checking the data extractions. 

### 2.5. Risk of Bias Assessment

The risk of bias assessment was done using the Cochrane Collaboration tool for the assessment of the risk of bias of randomized controlled trials [35].

### 2.6. Data Synthesis and Statistical Analysis

Details regarding year, study design, population, randomization, blinding, intervention and placebo agents, duration of follow up and baseline and follow up BMI and body weight and fat mass were systematically extracted from the included studies. The differences in the mean change from baseline in BMI/body weight/fat mass comprised the primary measure of treatment effect. The meta-analyses were performed by computing the difference in mean changes using the random effects model with an inverse variance in Revman 5.3 (The Cochrane Collaboration, 2014). The primary outcome measure was the difference in mean change in BMI/body weight and fat mass comparing pre-/pro-/syn-biotics to placebos.

The included studies reported a mix of change from baseline and baseline/final value. When a change from baseline was reported in a study without the standard deviation (SD) of change, the presented statistical analyses comparing change (e.g., confidence intervals (CI), standard errors (SE), *p*-values) were used to determine the SD [36]. When only the baseline, the final values and the SD were reported in a study, the mean change for each treatment group was obtained by subtracting the final mean from the baseline mean, which also allowed the imputation of the SD. Imputation includes three steps: (1) calculate the correlation coefficient (Corr) between the baseline and final values for each treatment group from the included studies that were reported in considerable detail; (2) take the mean of these Corrs and use the result as the imputed Corr; (3) impute the SD of mean change with the imputed Corr [36,37]. The chi-squared test (*X*^2^) and I^2^ were used to measure the heterogeneity among studies. Subgroup analyses stratified for the dose of probiotic and duration of treatment were performed to explore heterogeneity. Funnel plots and an Egger’s test of regression for funnel plot asymmetry were conducted to assess publication bias in the included studies.

## 3. Results

**Figure 2:**
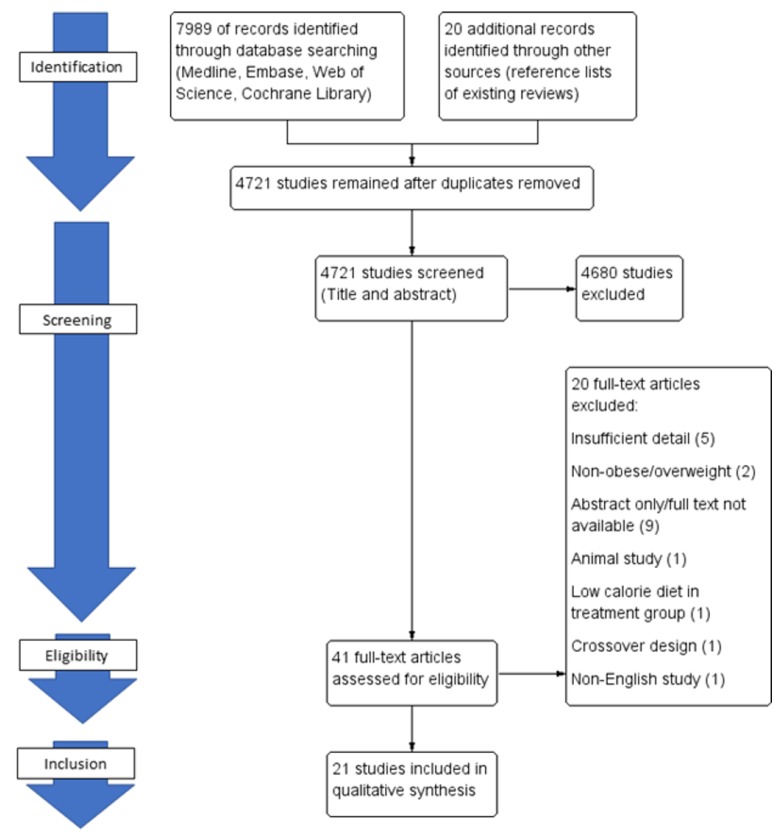
Study flow diagram.

A total of 8009 studies (7989 from library electronic search and 20 from references) were identified, from which 4721 studies remained after duplicates were removed (Figure 2). Two authors performed independent title and abstract screening to identify 41 articles, all of which underwent full-text screening. After the application of inclusion and exclusion criteria, 21 studies remained, thus yielding 33 study arms. Of these study arms, 22 were probiotic, six prebiotic and five synbiotic. The details of the studies with population, intervention, placebo used and duration are presented in Table 2. The majority of studies had healthy participants with a BMI ≥ 25. However, some studies included patients with diabetes mellitus or pre-diabetes, metabolic syndrome and non-alcoholic fatty liver disease (NAFLD). One study assessed the use of probiotics and prebiotics in patients post-Roux-en-Y gastric bypass [38]. The median duration of follow up in the studies was 12 weeks (range 2–24 weeks). Body composition and fat mass were determined using dual-energy X-ray absorptiometry (DXA) in the majority of studies (55%, *n* = 6) assessing fat mass, followed by bioelectric impedance (36%, *n* = 4) and body composition analyzer (9%, *n* = 1). 

### 3.1. Overall Effect of Pre-, Pro- or Syn-Biotics on Body Mass Index, Body Weight and Fat Mass

**Figure 3:**
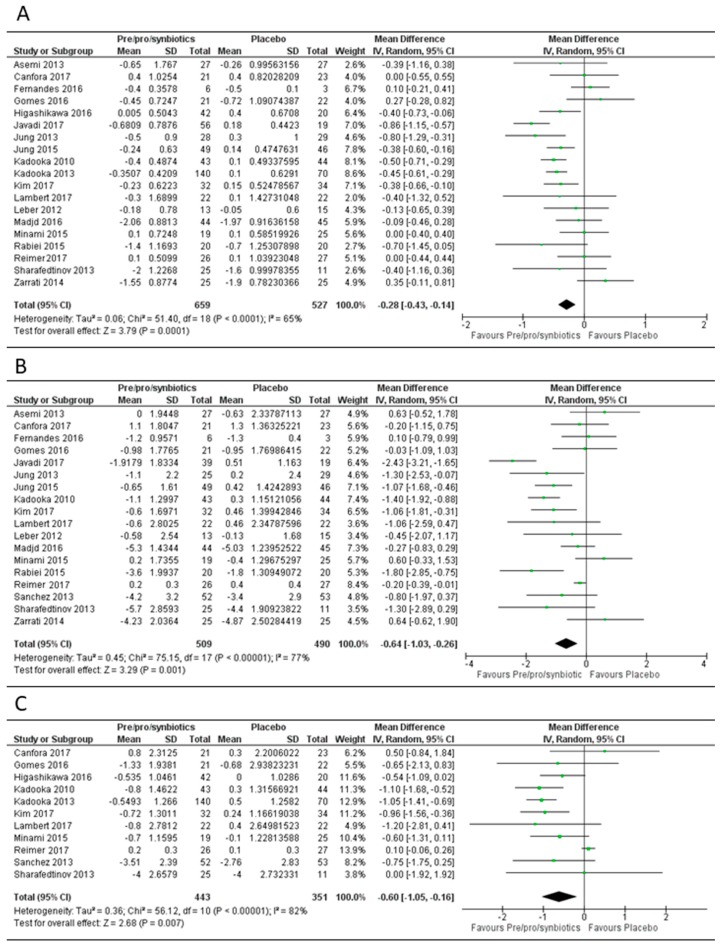
Forest plot of effect of any dietary modulation agent (prebiotic/probiotic/synbiotic) on: (**A**) body mass index (BMI); (**B**) body weight; (**C**) fat mass. Data synthesis using the random effects model for the mean differences amongst the included randomized controlled trials comparing all gut microbiome-modulating interventions (prebiotics/probiotics/synbiotics) vs. placebo showed significant differences for BMI in 19 studies (**A**) (overall mean difference (MD) = −0.28 (95% CI −0.43, −0.14), *p* < 0.001), body weight reduction in 18 studies (**B**) (MD = −0.64 kg (95% CI −1.03, −0.26), *p* < 0.001) and fat mass in 11 studies (**C**) (MD = −0.60 kg (95% CI −1.05, −0.16), *p* < 0.001). CI: Confidence intervals. SD: Standard deviation.

When any dietary modulation agent (prebiotic/probiotic/synbiotic) was compared to the placebo in 19 evaluable study arms (Figure 3), the mean difference in BMI was significant at −0.28 (95% CI −0.43, −0.14), *p* < 0.001. The mean difference for any agent was also significant for a reduction in body weight (−0.64 kg (95% CI −1.03, −0.26), and *p* < 0.001) in 18 evaluable study arms. Fat mass reduction was evaluated in 11 study arms and was statistically significant with a mean difference of −0.60 kg (95% CI −1.05, −0.16), *p* < 0.001. There was a significant heterogeneity among the study arms for BMI, weight and fat mass (I^2^ > 50%) for any dietary modulation agent (prebiotic/probiotic/synbiotic). When stratified and based on study duration (long duration > 12 weeks, short duration < 12 weeks), only studies with a duration >12 weeks showed a significant reduction in BMI and body weight (Figure S1). However, fat mass reduction was only significant in studies with a duration < 12 weeks (mean difference −0.83 kg (95% CI −1.26, −0.41), *p* < 0.001).

### 3.2. Effect of Probiotics on Body Mass Index, Weight and Fat Mass

**Figure 4:**
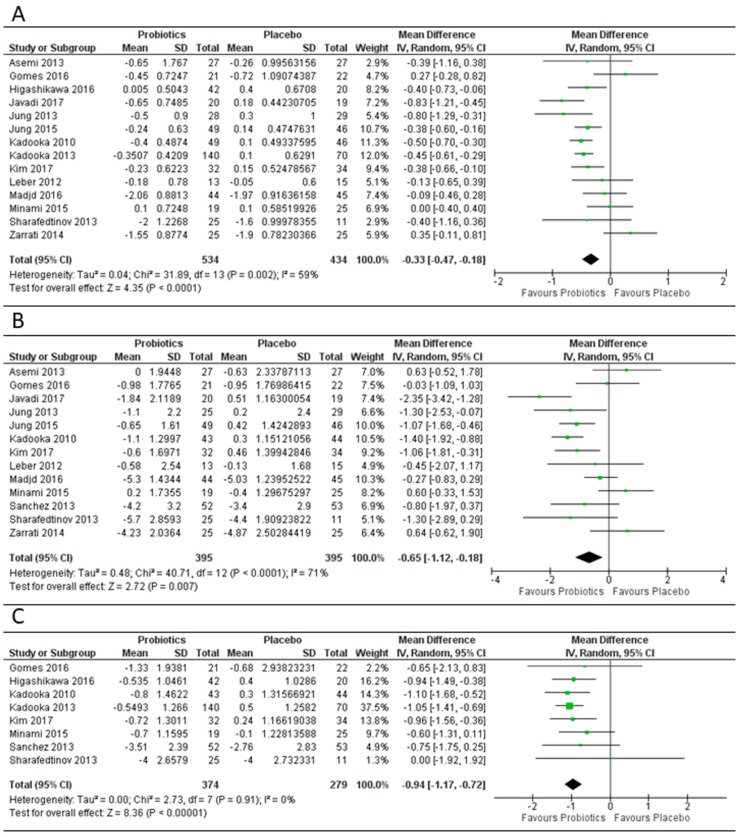
Forest plot of effect of probiotics on: (**A**) BMI; (**B**) body weight; (**C**) fat mass. Data synthesis using the random effects model for the mean differences amongst the included randomized controlled trials comparing probiotics vs. placebo showed significant differences for BMI in 14 study arms (**A**) (overall MD = −0.33 (95% CI −0.47, −0.18), *p* < 0.001), body weight reduction in 13 study arms (**B**) (MD = −0.65 kg (95% CI −1.12, −0.18), *p* < 0.01) and fat mass in 8 study arms (**C**) (MD = −0.94 kg (95% CI −1.17, −0.72), *p* < 0.001).

The effect of probiotics on BMI was evaluated in 14 study arms (Figure 4). Overall, probiotics led to a significant decrease in BMI compared to placebo (overall mean difference −0.33 (95% CI −0.47, −0.18), *p* < 0.001). The overall effect of probiotics on body weight reduction was significant, with an overall mean difference of −0.65 kg ((95% CI −1.12, −0.18), *p* < 0.01) in the 13 study arms that were evaluated. Overall, a significant reduction in fat mass was also seen, with eight study arms using probiotics, leading to a mean difference of −0.94 kg ((95% CI −1.17, −0.72), *p* < 0.001). There was significant heterogeneity in the study arms for BMI and body weight with I^2^ > 50%. However, for fat mass, the included probiotic study arms exhibited no heterogeneity (I^2^ = 0%). 

A subgroup analysis stratified by the dose of probiotic used (high dose >30 × 10^9^ colony forming units (CFUs), medium dose 1–30 × 10^9^ CFUs and low dose <1 × 10^9^ CFUs) showed a greater mean decrease in BMI for the study arms with high dose probiotics (mean difference −0.43 (95% CI −0.56, −0.30), *p* < 0.001) compared to low dose probiotics (−0.31 (95% CI −0.60, −0.02), *p* = 0.04) (Figure S2). However, for fat mass, studies using low dose probiotics had a greater mean decrease as compared to studies using high dose probiotics (mean difference −1.00 kg (95% CI −1.30, −0.71) vs. −0.88 kg (95% CI −1.23, −0.54)). Interestingly, when the studies were stratified by dose and duration (Supplementary Materials Figure S3), studies using low or medium probiotic doses for a duration >12 weeks showed a significant reduction in BMI (mean difference −0.38 (95% CI −0.59, −0.16), *p* < 0.001) and body weight (mean difference −0.98 kg (95% CI −1.54, −0.42), *p* < 0.001). When stratified by single vs. multispecies probiotic supplementation (Figure S4), single species studies showed a significant decrease in BMI (−0.41 (95% CI −0.56, −0.27), *p* < 0.001), body weight (−0.77 kg (95% CI −1.52, −0.03), *p* = 0.04) and fat mass (−0.95 kg (95% CI −1.19, −0.71), *p* < 0.001). Studies using *Lactobacillus* as a single strain showed a significant reduction in BMI (−0.47 (95% CI −0.59, −0.35), *p* < 0.001) and the greatest reduction in body weight (−1.25 kg (95% CI −1.66, −0.84), *p* < 0.001) and fat mass (−1.01 kg (−1.30, −0.72), *p* < 0.001) (Figure S5). However, for studies using multispecies probiotic supplementation, the reduction was only statistically significant for fat mass (−0.92 kg (95% CI −1.47, −0.36), *p* = 0.001). 

### 3.3. Effect of Prebiotics on Body Mass Index, Body Weight and Fat Mass

**Figure 5:**
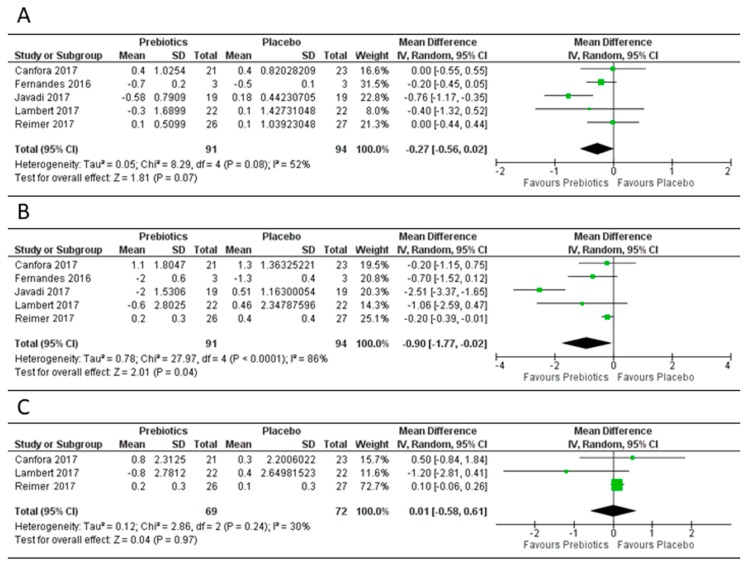
Forest plot of effect of prebiotics on: (**A**) BMI; (**B**) body weight; (**C**) fat mass. Data synthesis using the random effects model for the mean differences in body mass index (BMI), body weight and fat mass for the included randomized controlled trials of prebiotics versus placebo. Prebiotics intervention was found to trend towards a reduction in BMI in five studies with a MD = −0.27 ((95% CI −0.56, 0.02), *p* = 0.07) (Figure 5A), while the reduction in body weight in five studies was significant (MD = −0.90 kg (95% CI −1.77, −0.02), *p* = 0.04), Figure 5B. The prebiotics did not change the fat mass when compared to the placebo in three clinical trials, Figure 5C.

Prebiotics only had a marginal effect on the reduction of BMI, with a mean difference of −0.27 ((95% CI −0.56, 0.02), *p* = 0.07) in the five study arms evaluated (Figure 5). However, there was a significant reduction in body weight, with five study arms demonstrating a mean difference of −0.90 kg ((95% CI −1.77, −0.02), *p* = 0.04). Fat mass was evaluated in three prebiotic study arms with no significant change. Similar to probiotics, the prebiotic study arms for BMI and weight showed a significant heterogeneity (I^2^ > 50%); however, this was not significant for fat mass. 

### 3.4. Effect of Synbiotics on Body Mass Index, Weight and Fat Mass

**Figure 6:**
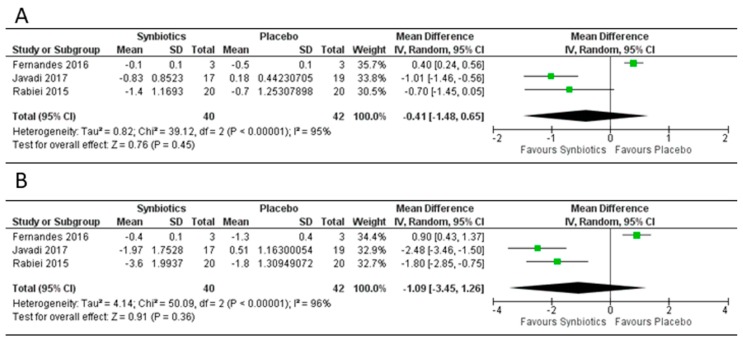
Forest plot of effect of synbiotics on: (**A**) BMI; (**B**) body weight. Data synthesis using the random effects model for the mean differences in BMI and body weight for the included randomized controlled trials of synbiotics versus placebo. There was no significant reduction in BMI or body weight in the three studies (Figure 6A,B).

There were only three study arms using synbiotics to assess change in BMI and weight. No studies evaluated change in fat mass (Figure 6), and there was no significant reduction in BMI or body weight. There was a significant heterogeneity in the symbiotic study arms for both BMI and weight (I^2^ > 50%). 

### 3.5. Risk of Bias Assessment

**Figure 7:**
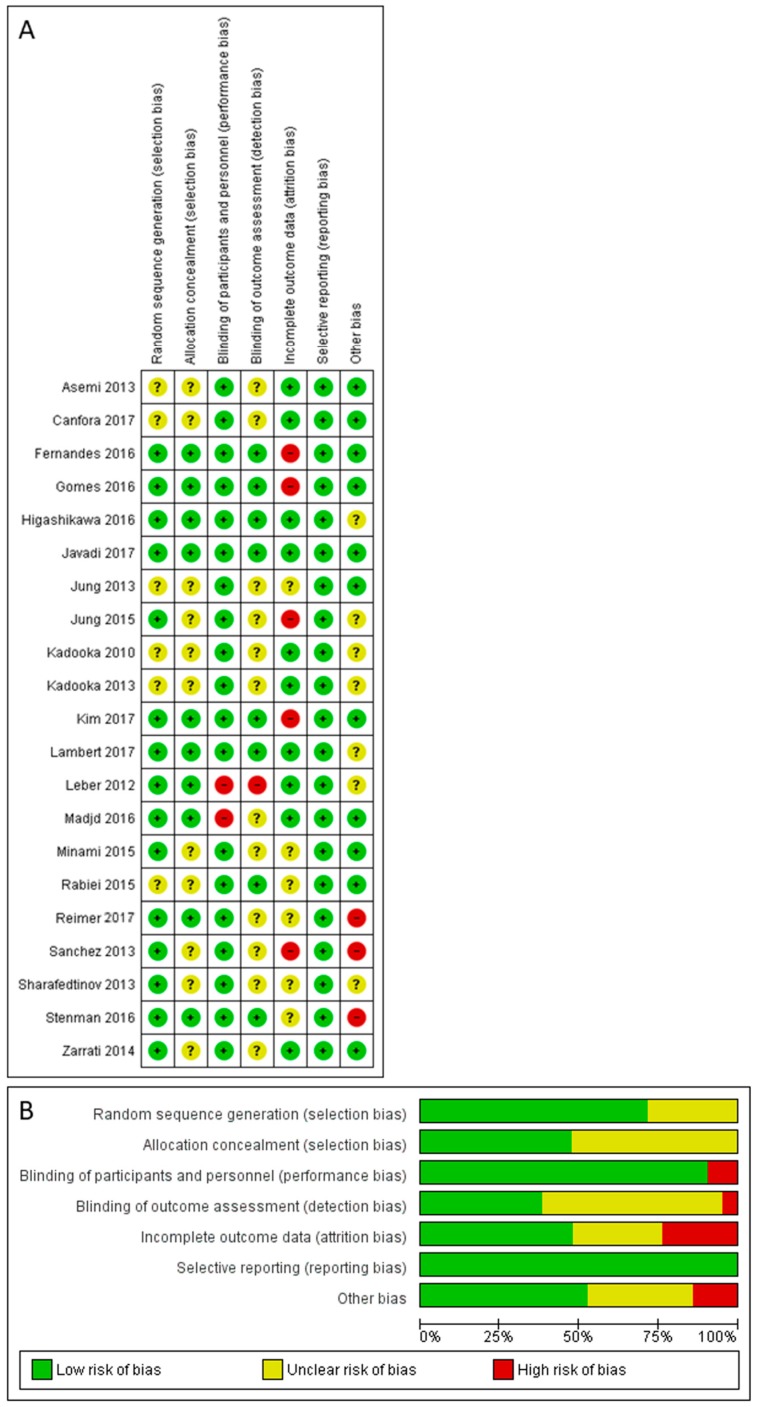
Risk of bias assessment. (**A**) Details of included studies; (**B**) overall summary.

The risk of bias among the studies was assessed using the Cochrane Collaboration tool for the assessment of the risk of bias of randomized controlled trials (Figure 7). There was no significant selection, performance, detection or reporting bias in the included studies. However, there was a moderate amount of attrition bias (24%) noted. Details regarding the risk of bias, including funding of the studies and potential conflicts of interest, are presented in Table S4.

### 3.6. Publication Bias Assessment

There was no significant publication bias identified in the analysis for the effect of any dietary modulation agent (prebiotic/probiotic/synbiotic) on BMI, weight and fat mass using regression for funnel plot asymmetry (Egger’s test *p* > 0.05). The funnel plots created for the visual analysis of publication bias are presented in Figure S6. 

## 4. Discussion

Dietary modulation of the gut microbiome for the treatment of obesity continues to remain an area of tremendous potential. Our findings in this study make a significant contribution to the evidence supporting the role of manipulating the gut microbiome to facilitate weight loss. In our meta-analysis of 21 studies, the use of probiotics led to significant reductions in BMI, body weight and fat mass when compared to the placebo. A subgroup analysis in five studies using single species probiotic agents revealed that *Lactobacillus* probiotics showed significant reductions in BMI and the greatest reductions in body weight and fat mass. A subgroup analysis also demonstrated significant body weight and BMI reductions in studies using low or medium probiotic doses of greater than 12 weeks in duration. Prebiotics support the growth of constituent probiotic microflora and are expected to contribute towards the amelioration of excess body weight; however, the use of prebiotics alone only led to a significant reduction in body weight in five studies, but not BMI or fat mass. Furthermore, synbiotics did not have any significant effect on weight loss or fat mass, although only three studies merited inclusion. 

Probiotic strains, such as *Lactobacillus* and *Bifidobacterium*, compete for nutrients with existing gut microbiota with negative effects [6]. Probiotics enrich the biofilm layer and prevent the adherence of pathogenic bacteria [59]. They have a key role in enhancing the intestinal epithelial barrier and reducing gut permeability, thereby reducing gut inflammation, preventing metabolic endotoxemia and improving insulin resistance [60,61,62]. The fat mass reduction effect seen with probiotics is possibly mediated through the deconjugation of bile acids, thereby making lipid absorption less effective from the diet [63]. Probiotics have also been shown to increase the levels of SCFAs in the diet by the fermentation of non-digestible carbohydrates [64]. Short chain fatty acids strengthen the intestinal barrier by promoting intestinal regeneration and mucin production, in addition to its positive effect on lipid metabolism [65,66]. Our findings with a higher effectiveness of probiotics over other agents for BMI, body weight and fat loss could be explained by the hypothesis that alteration of the gut microbiome is more predictable and successful with probiotics. 

Prebiotics have been shown to reduce appetite, improve insulin sensitivity and improve lipid metabolism in animal and human studies [62,67]. Prebiotic supplementation improves the gut barrier by allowing the proliferation of commensal gut flora and has been shown to reduce the levels of inflammatory cytokines in animal studies [67]. However, there are several factors, such as baseline gut microbiome profile (i.e., *Firmicutes/Bacteroidetes* ratio) and diet, which could influence the success of a prebiotic since its action is primarily the creation of a favorable environment for the growth of specific bacteria. The alteration of the gut microbiome is not restricted to dietary supplements alone. Alteration of the gut microbiome can also occur through the introduction of diets that favor different microbiota. A reduction in *Mollicute* predominance and an increase in *Bacteroidetes* has been demonstrated in obese humans with weight loss on a carbohydrate- or fat-restricted diet [68]. Similar results have been demonstrated in animal studies with the introduction of a high-fat, high-sugar diet, which leads to an increase in the normally less abundant *Mollicute* lineage of the *Firmicutes* to increase with an associated decrease in *Bacteroidetes* [69]. It is important to realize the bidirectional effect of the alteration of the gut microbiome on weight loss, as evidenced by the use of *Lactobacillus*, *Bifidobacterium* and other species in animal husbandry where it has been used for weight gain in livestock and where the careful selection of strains is of paramount importance in the treatment of obesity [70]. 

The finding of significant weight and BMI reduction in studies using low to medium dose probiotics over a long duration is highly relevant, since it suggests the potential effectiveness of long-term supplementation of over-the-counter probiotic supplements or fermented foods in the treatment of obesity compared to high dose probiotic supplements. Our findings of a single agent probiotic species such as *Lactobacillus* showing efficacy for reduction in BMI, body weight and fat mass can help guide consumers in a confusing and unregulated marketplace of probiotic supplements. However, further studies will be needed to confirm this finding. 

Our study has several strengths. We extensively reviewed the literature from several databases in order to make this meta-analysis the most comprehensive to date. Moreover, we restricted our population to adult humans with a BMI ≥ 25, thereby selecting the population of greatest interest, as compared to previous quantitative reviews, which included all studies regardless of BMI. We also performed separate analyses for probiotics, prebiotics and synbiotics, as well as a combined analysis to increase the specificity of our findings. However, this study also has a few limitations. While there was a large number of studies assessing the effects of probiotics, the analyses of prebiotic and synbiotic agents were restricted to a relatively small number. There was also significant heterogeneity in the studies with I^2^ > 50% for several of the analyses. It is possible that the use of different methods for fat mass and body composition determination contributed to heterogeneity in the analysis of the effect of dietary interventions on fat mass. This was offset to some extent by the use of a random effects model of meta-analysis. We also performed a subgroup analysis stratified by dose and duration of supplementation to decrease heterogeneity and increase interpretability. However, regional variations in local food, differences in baseline gut microbiota in different geographical regions of the world and absence of baseline and post-intervention microbiome data for all the studies make any pooled analyses of dietary interventions for modulating the gut microbiome inherently heterogeneous. Moreover, the sustainability of probiotic interventions on the ecology of the gut microbiome may also vary, and further studies are needed to assess the sustainability of weight loss induced by these agents. 

## 5. Conclusions

In conclusion, dietary agents for modulation of the gut microbiome are essential tools in the treatment of obesity and lead to significant reductions in BMI, body weight and fat mass when compared to placebo. Further studies are needed to determine the ideal formulation for supplementation and to identify specific populations of overweight patients who would benefit most from gut microbiome modulation, in addition to assessing the durability of this effect. 

## Figures and Tables

**Table 1:** Search terms.

**Medline (PubMed)**
Search hits 1892 (“Probiotics”[Mesh] OR probiotic*[tw] OR “Prebiotics”[Mesh] OR prebiotic*[tw] OR “Synbiotics”[Mesh] OR synbiotic*[tw] OR “Lactobacillus”[Mesh] OR lactobacillus[tw] OR “Yeast, Dried”[Mesh] OR “dried yeast”[tw]) AND (“Body Weight Changes”[Mesh] OR weight change*[tw] OR weight gain*[tw] OR weight loss*[tw] OR weight regulation*[tw] OR “weight modification”[tw] OR “Obesity”[Mesh] or obes*[tw] OR “Overweight”[Mesh] OR overweight[tw])
**Embase**
Search hits 3126 (‘probiotic agent’/exp OR probiotic*:ti,ab OR ‘prebiotic agent’/exp OR prebiotic*:ti,ab OR ‘synbiotic agent’/exp OR synbiotic*:ti,ab OR ‘Lactobacillus’/exp OR lactobacillus:ti,ab OR ‘dried yeast’/exp OR ‘dried yeast’:ti,ab) AND (‘weight change’/exp OR ‘weight change*’:ti,ab OR ‘weight gain*’:ti,ab OR ‘weight loss*’:ti,ab OR ‘weight regulation*’:ti,ab OR ‘weight modification’:ti,ab OR ‘obesity’/exp or obes*:ti,ab OR overweight:ti,ab)
**Web of Science**
Search hits 2694 #1. TS = (probiotic* OR prebiotic* OR synbiotic* OR lactobacillus OR “dried yeast”) #2. TS = (“weight change*” OR “weight gain*” OR “weight loss*” OR “weight regulation*” OR “weight modification” OR obes* OR overweight) #3. #1 AND #2
**Cochrane Library**
Search hits 277 #1 probiotic* or prebiotic* or synbiotic* or lactobacillus or “dried yeast”:ti,ab,kw #2 “weight change*” or “weight gain*” or “weight loss*” or “weight regulation*” or “weight modification” or obes* or overweight:ti,ab,kw #3 MeSH descriptor: [Probiotics] explode all trees #4 MeSH descriptor: [Prebiotics] explode all trees #5 MeSH descriptor: [Synbiotics] explode all trees #6 MeSH descriptor: [Lactobacillus] explode all trees #7 MeSH descriptor: [Yeast, Dried] explode all trees #8 #1 or #3 or #4 or #5 or #6 or #7 #9 MeSH descriptor: [Body Weight Changes] explode all trees #10 MeSH descriptor: [Obesity] explode all trees #11 MeSH descriptor: [Overweight] explode all trees #12 #2 or #9 or #10 or #11 #13 #8 and #12

**Table 2:** Included study arms.

Author	Country	Year	Randomized	Blinding	Placebo Control	Overweight or Obese	Population	Probiotic/Prebiotic/Synbiotic	Single or Multi-Strain Probiotic/Prebiotic Agent Used	Daily Dose (in Billions, 10^9^) CFU/Dose of Prebiotic in Grams	Duration (Weeks)
Asemi [39]	Iran	2013	Y	Y	Y	Both	T2DM	Probiotic	Multi	39.2 × 10^9^	8
Canfora [40]	Netherlands	2017	Y	Y	Y	Both	Prediabetic	Prebiotic	GOS	15 g	12
Fernandes [38]	Brazil	2016	Y	Y	Y	Obese	RYGB	Prebiotic	FOS	6 g	2 ^#^
Fernandes [38]	Brazil	2016	Y	Y	Y	Obese	RYGB	Synbiotic	Multi + FOS	4 × 10^9^ + 6 g	2 ^#^
Gomes [41]	Brazil	2016	Y	Y	Y	Both	Healthy women	Probiotic	Multi	20.0 × 10^9^	8
Higashikawa [42]	Japan	2016	Y	Y	Y	Overweight	Healthy	Probiotic	Single (*Pediococcus pentosaceus* LP28, heat killed)	100 × 10^9^	12
Higashikawa [42]	Japan	2016	Y	Y	Y	Overweight	Healthy	Probiotic	Single (*P. pentosaceus* LP28, living)	100 × 10^9^	12
Javadi [43]	Iran	2017	Y	Y	Y	Both	NAFLD	Probiotic	Multi	0.02 × 10^9^	12
Javadi [43]	Iran	2017	Y	Y	Y	Both	NAFLD	Prebiotic	Inulin	10 g	12
Javadi [43]	Iran	2017	Y	Y	Y	Both	NAFLD	Synbiotic	Multi + inulin	0.02 × 10^9^ + 10 g	12
Jung [44]	Korea	2015	Y	Y	Y	Overweight	Healthy	Probiotic	Multi	10 × 10^9^	12
Jung [45]	Korea	2013	Y	Y	Y	Both	Healthy	Probiotic	Single (*Lactobacillus gasseri* BNR17)	60 × 10^9^	12
Kadooka [46]	Japan	2010	Y	Y	Y	Overweight	Healthy	Probiotic	Single (*L. gasseri* SBT2055)	50 × 10^9^	12
Kadooka [46]	Japan	2010	Y	Y	Y	Overweight	Healthy	Probiotic	Single *(L. gasseri* SBT2055)	50 × 10^9^	8
Kadooka [46]	Japan	2010	Y	Y	Y	Overweight	Healthy	Probiotic	Single (*L. gasseri* SBT2055)	50 × 10^9^	4
Kadooka [47]	Japan	2013	Y	Y	Y	Overweight	Healthy	Probiotic	Single * (*L*.*gasseri* SBT2055)	0.08 × 10^9^	12
Kadooka [47]	Japan	2013	Y	Y	Y	Overweight	Healthy	Probiotic	Single * (*L.gasseri* SBT2055)	0.007 × 10^9^	12
Kim [48]	Korea	2017	Y	Y	Y	Overweight	Healthy	Probiotic	Multi	5 × 10^9^	12
Lambert [49]	Canada	2017	Y	Y	Y	Both	Healthy	Prebiotic	Yellow pea fiber	5 g	12
Leber [50]	Austria	2012	Y	N	N	Both	Metabolic syndrome	Probiotic	Single (*L. casei* Shirota)	19.5 × 10^9^	12
Madjd [51]	Iran	2016	Y	Y	Y	Both	Healthy women	Probiotic	Multi	0.01 × 10^9^	12
Minami [52]	Japan	2015	Y	Y	Y	Overweight	Healthy	Probiotic	Single (*Bifidobacterium breve* B-3)	50 × 10^9^	12
Minami [52]	Japan	2015	Y	Y	Y	Overweight	Healthy	Probiotic	Single (*B. breve* B-3)	50 × 10^9^	8
Minami [52]	Japan	2015	Y	Y	Y	Overweight	Healthy	Probiotic	Single (*B. breve* B-3)	50 × 10^9^	4
Rabiei [53]	Iran	2015	Y	Y	Y	Both	Metabolic syndrome	Synbiotic	Multi + FOS	0.2 × 10^9^ + NA	12
Rabiei [53]	Iran	2015	Y	Y	Y	Both	Metabolic syndrome	Synbiotic	Multi + FOS	0.2 × 10^9^+NA	6
Reimer [54]	Canada	2017	Y	Y	Y	Both	Healthy	Prebiotic	Oligofructose + Inulin	6 g + 2 g	12
Sanchez [55]	Canada	2013	Y	Y	Y	Both	Healthy	Probiotic	Single (*L. rhamnosus* CGMCC1.3724)	0.32 × 10^9^	12
Sharafedtinov [56]	Estonia	2013	Y	Y	Y	Obese	Metabolic syndrome	Probiotic	Single (*L. plantarum* TENSIA)	150 × 10^9^	3
Stenman [57]	Finland	2016	Y	Y	Y	Both	Healthy	Probiotic	Single (*B. animalis* ssp. *lactis* 420)	10 × 10^9^	24
Stenman [57]	Finland	2016	Y	Y	Y	Both	Healthy	Prebiotic	Polydextrose	12 g	24
Stenman [57]	Finland	2016	Y	Y	Y	Both	Healthy	Synbiotic	Single + polydextrose	10 × 10^9^ + 12 g	24
Zarrati [58]	Iran	2014	Y	Y	Y	Both	Healthy	Probiotic	Multi	0.03 × 10^9^	8

^#^ Fifteen days. * Single strain of *L. gasseri* 2055 added to starter yogurt cultures of multi-strain bacteria. + Dose of bacteria added to cheese before cheese coagulation (renneting). T2DM, type 2 diabetes mellitus; FOS, fructooligosaccharide; GOS, galactooligosaccharide; RYGB, Roux-en-Y gastric bypass NAFLD; non-alcoholic fatty liver disease; NA not available. CFU: Colony forming units.

## Supplementary Materials

The following are available online at http://www.mdpi.com/2073-4425/9/3/167/s1, Table S1: Study arms for BMI analysis, Table S2: Study arms for body weight analysis, Table S3: Study arms for fat mass analysis, Table S4: Risk of bias assessment details, Table S5: Details of dietary agents and placebo for included studies, Figure S1: Subgroup analysis of the effect of the duration of supplementation of the dietary modulation agent on: (A) BMI; (B) body weight; (C) fat mass, Figure S2: Subgroup analysis: forest plot of effect of probiotic dosing on: (A) BMI; (B) body weight; (C) fat mass, Figure S3: Subgroup analysis: forest plot of effect of probiotic dosing and duration on: (A) BMI; (B) body weight; (C) fat mass, Figure S4: Subgroup analysis: forest plot of the effect of single species vs. multi-species probiotic supplementation: (A1) Effect of multi-species probiotic supplementation on BMI; (A2) effect of single species probiotic supplementation on BMI (B1) Effect of multi-species probiotic supplementation on body weight; (B2) effect of single species supplementation on body weight; (C1) effect of multi-species probiotic supplementation on fat mass; (C2) effect of single species supplementation on fat mass; Figure S5: Subgroup analysis: forest plot of the effect of *Lactobacillus* supplementation on: (A) BMI; (B) body weight; (C) fat mass, Figure S6: Funnel plot for publication bias for studies using any dietary agent (prebiotic/probiotic/synbiotic) on: (A) BMI; (B) body weight; (C) fat mass, Figure S7: Forest plot of the effect of any dietary modulation agent (prebiotic/probiotic/synbiotic) on: (A) BMI and (B) body weight excluding studies involving subjects with NAFLD, T2DM and RYGB.
