# Supplementary material for: Dietary Alteration of the Gut Microbiome and Its Impact on Weight and Fat Mass: A Systematic Review and Meta-Analysis
Source: Genes (Basel). 2018 Mar 16;9(3):167. doi: 10.3390/genes9030167 (PMC5867888; doi:10.3390/genes9030167)
Supplement: Supplementary file 1 [file genes-09-00167-s001.zip › Table S1.pdf]

Last Author	Year	Treatment group	Control group	Baseline BMI control	SD	Post intervention BMI control group	SD	Baseline BMI treatment	SD	Post intervention BMI treatment group	SD	Change in BMI control	SD	Change in BMI treatment	SD	Difference in BMI change(trt-control)	Control group correlation r between baseline and followup BMI	Treatment group correlation r between baseline and followup BMI
Asemi	2013	27	27	30.17	4.23	29.91	4.27	31.61	6.36	30.96	6.39	-0.26	0.995631565	-0.65	1.76702	-0.39	0.972603346	0.961596602
Canfora	2017	21	23	32.3	3.5	32.7	3.3	33.3	3.7	33.7	3.7	0.4	0.820282092	0.4	1.02542	0	0.972603346	0.961596602
Fernandes	2016	3	3	38.2	1.4	37.7	1.4	40.2	4.5	39.5	4.4	-0.5	0.1	-0.7	0.2	-0.2	0.99744898	0.999242424
Fernandes	2016	3	3	38.2	1.4	37.7	1.4	41.6	5.2	41.5	5.2	-0.5	0.1	-0.1	0.1	0.4	0.99744898	0.999815089
Gomes	2016	21	22	33.34	4.69	32.61	4.53	31.7	3.9	31.24	3.96	-0.72	1.090743869	-0.45	0.72475	0.27	0.972603346	0.983111172
Higashikawa	2016	21	20	27.37	1.43108	27.77	1.78885	26.84	1.2373	26.91	1.28312	0.4	0.670820393	0.07	0.54991	-0.33	0.937109375	0.90542328
Higashikawa	2016	21	20	27.37	1.43108	27.77	1.78885	27.1	1.14564	27.04	1.37477	0.4	0.670820393	-0.06	0.45826	-0.45	0.937109375	0.95
Javadi	2017	20	19	30.38	2.88	30.56	2.88	29.91	3.88	29.26	3.59	0.18	0.44230705	-0.65	0.74854	-0.83	0.98820677	0.982905925
Javadi	2017	19	19	30.38	2.88	30.56	2.88	30.96	4.39	30.38	4.63	0.18	0.44230705	-0.58	0.7909	-0.76	0.98820677	0.986029552
Javadi	2017	17	19	30.38	2.88	30.56	2.88	32.3	4.78	31.47	4.58	0.18	0.44230705	-0.83	0.85235	-1.01	0.98820677	0.984321074
Jung	2013	28	29	29.6	3.6	29.3	4	28.6	2.2	28.1	1.9	0.3	1	-0.5	0.9	-0.8	0.970833333	0.913875598
Jung	2015	49	46	27	1.49211	27.1	1.49211	27	1.47	26.8	1.61	0.14	0.474763099	-0.24	0.63	-0.38	0.949380165	0.920289855
Kadooka	2010	43	44	27.2	1.64459	27.3	1.80905	27.5	1.62467	27.2	1.62467	0.1	0.493375954	-0.4	0.4874	-0.5	0.963636364	0.955
Kadooka	2013	69	70	NA	NA	NA	NA	NA	NA	NA	NA	0.1	0.629084901	-0.3	0.41627	-0.4	NA	NA
Kadooka	2013	71	70	NA	NA	NA	NA	NA	NA	NA	NA	0.1	0.629084901	-0.4	0.42248	-0.5	NA	NA
Kim	2017	32	34	27.1	1.57436	27.2	1.63267	26.6	1.30108	26.4	1.41421	0.15	0.524785671	-0.23	0.62225	-0.38	0.947089947	0.89826087
Lambert	2017	22	22	33.3	6.09754	33.4	6.09754	33.1	6.09754	32.8	6.09754	0.1	1.427310478	-0.3	1.68987	-0.4	0.972603346	0.961596602
Leber	2012	13	15	31.6	3.6	31.6	4	35.4	5.3	35.3	5.5	-0.05	0.6	-0.18	0.78	-0.13	0.993055556	0.990250429
Madjd	2016	44	45	32.05	3.94	30.08	3.86	32.14	3.2	30.08	3.15	-1.97	0.916361582	-2.06	0.88131	-0.09	0.972603346	0.961596602
Minami	2015	19	25	27.7	2.5	27.8	2.5	27.1	2.61534	27.2	2.61534	0.1	0.585199262	0.1	0.72482	0	0.972603346	0.961596602
Rabiei	2015	20	20	32.7	5.39	32	5.23	32	4.08	30.6	4.26	-0.7	1.253078976	-1.4	1.16934	-0.7	0.972603346	0.961596602
Reimer	2017	26	27	31.3	6.23538	31.4	6.23538	31.9	4.58912	31.9	4.58912	0.1	1.039230485	0.1	0.5099	0	0.986111111	0.99382716
Sharafedtinov	2013	25	11	36.3	4.3	34.7	4.2	37.7	4.3	35.7	3.8	-1.6	0.99978355	-2	1.22679	-0.4	0.972603346	0.961596602
Zarrati	2014	25	25	33.9	3.365	32	3.265	33.8	3.175	32.25	3.155	-1.9	0.782303655	-1.55	0.87737	0.35	0.972603346	0.961596602

Calculated from SEM, 95%CI or p value (blue) — Imputed (yellow)
