# Supplementary material for: Dietary Alteration of the Gut Microbiome and Its Impact on Weight and Fat Mass: A Systematic Review and Meta-Analysis
Source: Genes (Basel). 2018 Mar 16;9(3):167. doi: 10.3390/genes9030167 (PMC5867888; doi:10.3390/genes9030167)
Supplement: Supplementary file 1 [file genes-09-00167-s001.zip › Table S2.pdf]

Last Author	Year	Treatment group	Control group	Baseline weight control	SD	Post intervention weight control group	SD	Baseline weight treatment	SD	Post intervention weight treatment group	SD	Change in weight control	SD	Change in weight treatment	SD	Difference in weight change(trt-control)	Control group correlation r between baseline and followup weight	Treatment group correlation r between baseline and followup weight
Asemi	2013	27	27	73.03	13.3	72.4	11.42	73.48	13.37	73.48	13.37	-0.63	2.337871129	0	1.9448	0.63	0.993642464	0.989420859
Canfora	2017	21	23	96.9	11.5	98.2	11	98.4	11.9	99.5	12.3	1.3	1.363252214	1.1	1.8047	-0.2	0.993642464	0.989420859
Fernandes	2016	3	3	103	8.7	101.7	8.7	109.5	19.3	107.4	18.8	-1.3	0.4	-2	0.6	-0.7	0.998943057	0.999848418
Fernandes	2016	3	3	103	8.7	101.7	8.7	114.1	19.9	113.9	19.8	-1.3	0.4	-0.4	0.1	0.9	0.998943057	1
Gomes	2016	21	22	83.55	13.44	82.59	13	76.58	9.94	75.6	10.24	-0.95	1.769864152	-0.98	1.7765	-0.03	0.991589918	0.984939351
Javadi	2017	20	19	86	11.98	86.51	12.05	86.92	12.37	85.08	12.25	0.51	1.163000542	-1.84	2.1189	-2.35	0.995332226	0.985232424
Javadi	2017	19	19	86	11.98	86.51	12.05	88.45	10.36	86.45	10.54	0.51	1.163000542	-2	1.5306	-2.51	0.995332226	0.989420859
Javadi	2017	17	19	86	11.98	86.51	12.05	89.88	11.92	87.91	12.08	0.51	1.163000542	-1.97	1.7528	-2.48	0.995332226	0.989420859
Jung	2013	25	29	77.4	11	77.2	11.6	77.2	11	76.1	10	0.2	2.4	-1.1	2.2	-1.3	0.978840125	0.982545455
Jung	2015	49	46	73.5	9.63091	73.9	10.1057	73.5	8.96	72.9	9.1	0.42	1.424289296	-0.65	1.61	-1.07	0.990736364	0.98422476
Kadooka	2010	43	44	77.1	10.3609	77.4	10.6898	76.9	9.91049	75.8	9.74803	0.3	1.151210559	-1.1	1.2997	-1.4	0.994505495	0.991393443
Kim	2017	32	34	73.2	10.0292	73.6	10.6123	71.9	8.0893	71.3	8.48528	0.46	1.399428455	-0.6	1.6971	-1.06	0.992397138	0.98016317
Lambert	2017	22	22	93.7	20.6378	94.1	20.1688	92.3	19.2307	91.5	18.7617	0.46	2.347875963	-0.6	2.8025	-1.06	0.993642464	0.989420859
Leber	2012	13	15	90.9	14.3	90.8	15.1	108.3	15.9	107.8	16.6	-0.13	1.68	-0.58	2.54	-0.45	0.99494651	0.988706524
Madjd	2016	44	45	82.45	11.01	77.42	10.94	82.69	9.87	77.39	9.68	-5.03	1.239525225	-5.3	1.4344	-0.27	0.993642464	0.989420859
Minami	2015	19	25	71.2	11.5	70.8	11.5	68.9	11.769	69.1	11.3331	-0.4	1.296752969	0.2	1.7355	0.6	0.993642464	0.989420859
Rabiei	2015	20	20	84	11.6	82.2	11.43	81.3	13.5	77.7	13.73	-1.8	1.309490724	-3.6	1.9937	-1.8	0.993642464	0.989420859
Reimer	2017	26	27	90.3	4.8	90.7	4.9	92.4	3.8	92.5	3.7	0.4	0.4	0.2	0.3	-0.2	0.996811224	0.99715505
Sanchez	2013	52	53	NA	NA	NA	NA	NA	NA	NA	NA	-3.4	2.9	-4.2	3.2	-0.8	NA	NA
Sharafedtinov	2013	25	11	102.4	14.4	98	13.3	105.6	16.2	99.9	14.4	-4.4	1.909238218	-5.7	2.8593	-1.3	0.993642464	0.989420859
Zarrati	2014	25	25	87	22	82.13	21.5	90.63	14	86.4	14	-4.87	2.502844186	-4.23	2.0364	0.64	0.993642464	0.989420859

Calculated from SEM, 95%CI or p value

Imputed
