# Supplementary material for: Dietary Alteration of the Gut Microbiome and Its Impact on Weight and Fat Mass: A Systematic Review and Meta-Analysis
Source: Genes (Basel). 2018 Mar 16;9(3):167. doi: 10.3390/genes9030167 (PMC5867888; doi:10.3390/genes9030167)
Supplement: Supplementary file 1 [file genes-09-00167-s001.zip › Table S3.pdf]

Last Author	Year	Treatment group	Control group	Baseline fat mass control	SD	Post intervention fat mass control group	SD	Baseline fat mass treatment	SD	Post intervention fat mass treatment group	SD	Change in fat mass control	SD	Change in fat mass treatment	SD	Difference in fat mass change(trt-control)	Control group correlation r between baseline and followup fat mass	Treatment group correlation r between baseline and followup fat mass
Canfora	2017	21	23	36	9	36.3	8.9	36.3	8.9	37.1	8.9	0.3	2.200602197	0.8	2.3125	0.5	0.969833645	0.966244774
Gomes	2016	21	22	39.73	8.23	39.05	7.35	36.3	7.32	34.96	7.52	-0.68	2.938232308	-1.33	1.9381	-0.65	0.935040964	0.966244774
Higashikawa	2016	21	20	23.81	4.51686	24.21	4.96407	23.31	3.62023	23.01	3.98684	0.4	1.02859127	-0.3	1.0082	-0.7	0.980867006	0.969445657
Higashikawa	2016	21	20	23.81	4.51686	24.21	4.96407	23.86	4.76588	23.09	4.53675	0.4	1.02859127	-0.77	1.054	-1.17	0.980867006	0.975524476
Kadooka	2010	43	44	22.9	4.93376	23.2	4.60484	23.5	5.68635	22.7	5.19895	0.3	1.31566921	-0.8	1.4622	-1.1	0.964285714	0.967857143
Kadooka	2013	69	70	NA	NA	NA	NA	NA	NA	NA	NA	0.5	1.258169803	-0.6	1.457	-1.1	NA	NA
Kadooka	2013	71	70	NA	NA	NA	NA	NA	NA	NA	NA	0.5	1.258169803	-0.5	1.0562	-1	NA	NA
Kim	2017	32	34	22.99	4.43152	23.24	4.54814	22.16	3.33754	21.44	3.50725	0.24	1.166190379	-0.72	1.3011	-0.96	0.96659919	0.928922909
Lambert	2017	22	22	33.4	10.788	33.8	10.788	34.8	10.788	34	10.3189	0.4	2.649815226	-0.8	2.7812	-1.2	0.969833645	0.966244774
Minami	2015	19	25	23.5	5	23.4	5	21.5	4.3589	20.8	3.92301	-0.1	1.228135879	-0.7	1.1595	-0.6	0.969833645	0.966244774
Reimer	2017	26	27	27.9	2.2	28	2.1	29	2	29.3	1.9	0.1	0.3	0.2	0.3	0.1	0.991341991	0.989473684
Sanchez	2013	52	53	NA	NA	NA	NA	NA	NA	NA	NA	-2.76	2.83	-3.51	2.39	-0.75	NA	NA
Sharafedtinov	2013	25	11	46.4	7.9	42.4	9.6	46.7	10.3	42.7	9.8	-4	2.732330999	-4	2.6579	0	0.969833645	0.966244774

Calculated from SEM, 95%CI or p value

Imputed
