# Supplementary material for: Dietary Alteration of the Gut Microbiome and Its Impact on Weight and Fat Mass: A Systematic Review and Meta-Analysis
Source: Genes (Basel). 2018 Mar 16;9(3):167. doi: 10.3390/genes9030167 (PMC5867888; doi:10.3390/genes9030167)
Supplement: Supplementary file 1 [file genes-09-00167-s001.zip › Table S4.pdf]

Risk table for Microbiome interventional study

Color code	Low	Unclear	High

Author(s)	Random sequence Generation	Allocation Concealment	Blinding of Participants and Personnel	Blinding of outcome assessment	Incomplete Outcome Data	Selective Reporting	Other Potential Bias (Industry fundings, conflicts of interest)
Asemi 2013	NR	NR	Yes	NR	No. 3/30 lost to followup in each group with similar reasons.	None	Zist Takhmir Company provided probiotic for the study not involved in data handling
Canfora 2017	NR	NR	Yes	NR	No. 1/22 in treatment group and 1/24 in control group dropped out of the study because of the use of antibiotics during the study period (1 for a wound infection after a bicycle fall and 1 for a lower respiratory tract infection)	None	The research was funded by TI Food and Nutrition, a public–private partnership on precompetitive research in food and nutrition. The funders had no role in the study design, data collection and analysis, decision to publish, or preparation of the manuscript.
Fernandes 2016	The participants were allocated randomly into the treatment arms using a randomization list generated by the Research Randomizer program, consisting of randomly permuted blocks with 3 individuals each.	All individuals evaluated were assigned to the treatment arm according to the randomization number. A copy of the randomization sequence was kept in a locked cabinet apart from the study personnel.	Study participants and investigators were blinded to the consumption and the distribution of supplementation. respectively.	Laboratory technicians who performed blood collection were blinded to the distribution of supplementation.	31 randomized, 18 completed 13 lost to follow up, details provided , non-compliance the central issue.	None	FarmaNutricao provide drug-not involved in data handling
Gomes 2016	The randomization list was provided by an independent research group not involved in the study using the Excel program 1997 to 2003 (Microsoft, Redmond, WA, EUA).	The blinding code was provided to the investigators after the statistical analyses were completed.	Yes	Yes	9/30 and 8/30 lost to followup in treatment and control group respectively.	None	Supported by the Fundac¸~ao de Amparo a Pesquisa do Estado de Goi as (FAPEG),
Higashikawa 2016	The allocation sequence was generated by using a Microsoft Excel randomization function.	Randomization assignments were carried out by nonclinical staff who had no other involvement in the trial.	Yes	The subjects and outcome assessors were blinded to treatment allocation.	4/42 in treatment group(2 for constipation, 1 for hive, 1 unable to contact) and 0/20 in control group dropped out of study. Details provided.	None	University and co-investigators have patent on probiotic LP28 used in the study
Javadi 2017	Computer-generated randomization scheme with block sizes of four and eight and an allocation ratio of 1:1:1:1.	The allocation was performed by an investigator with no clinical involvement in the study.	Yes	The main investigator and statistical data analyst remained blinded until the end of the analysis.	1/21(hepatitis), 2/21(personal reasons),4/21(2 travel 2 diabetes),2/21 lost to followup(hepatitis and diabetes). Was not intention to treat analysis.	None	No, Funded by Federal grant Iranian government
Jung 2013	NR	NR		NR	6/31 in treatment group and 2/31 in control group refused study procedures during the study.	None	No
Jung 2015	The randomization was performed via computergenerated block randomization	NR	Yes	NR	25 of 120 subjects (14 placebo and 11 probiotic) dropped out [one participant no longer satisfied the screening criteria (placebo); two participants developed severe colds that required antibiotics (placebo); and eight participants wanted to drop out voluntarily (4 placebo and 4 probiotic); seven participants denied CT, DEXA or anthropometric measurements (4 placebo and 3 probiotic); and seven participants failed to follow their habitual eating or physical activity patterns (3 placebo and 4 probiotic)].	None	Funded through Korean government sponsored programs
Kadooka 2010	NR	NR	Yes	NR	None of the 87 patients withdrew from the study. 10 centers. 12 wks		Yes, prinicpal investigator is employed by the maker of the probiotic
Kadooka 2013	NR	NR	Yes	NR	No subjects dropped out due to adverse events after the initiation of the study.	None	Yes, prinicpal investigator is employed by the maker of the probiotic
Kim 2017	The randomization was conducted via computer-generated block randomization	Only the product provider retained the control and probiotic group list, which was disclosed after the research and all laboratory analyses were completed.	Yes	Only the product provider retained the control and probiotic group list, which was disclosed after the research and all laboratory analyses were completed.	95/120 individuals were included in the final analysis. The reasons for lost-to-followup unknown	None	The product (powder) provider (Korea Yakult Co., Ltd, Yongin, Gyeonggi, Korea) retained thecontrol and probiotic group list, which was disclosed after the research and all laboratory analyses were completed
Lambert 2017	After screening, participants were randomly assigned using computer generated numbers (and stratified according to age, sex, and BMI) to either placebo (PL) or pea fiber (PF; 15 g/ d yellow pea fiber) for 12 weeks.	The randomization sequence was generated by an investigator not involved in recruiting participants and sequences were not revealed to study staff.	Yes	Participants and research staff were blinded to treatments.	53 adults were recruited and, of these, 47 completed the study. Six individuals dropped out (2 in the PL group and 4 in the PF group) for reasons including new pregnancy, employment-related, or undisclosed. Three subjects (all in the PF group) were excluded from analysis due to worsening of preexisting disease unrelated to the study or marked change in lifestyle due to family member illness, such that data analysis is presented on 44 subjects (22 in the PL group and 22 in the PF group).	None	Funding agency (Biosolutions) and other agencies) had no role in study design, data collection, analysis, and interpretation, or manuscript preparation.
Leber 2012	Patients were randomized into two groups (Randomizer, https://www.randomizer.at, Institute for Medical Informatics, Statistics and Documentation of the Medical Universitv of Graz. Graz. Austira).	Patients were randomized into two groups (Randomizer, https://www.randomizer.at, Institute for Medical Informatics, Statistics and Documentation of the Medical	Open-label study	Open-label study	30 patients were finally included, whereof 28 finished the study (2 dropped out due to withdrawal of informed consent).	None	Yakult provided the probiotic drink and supported the study in part.
Madjd 2016	Computer-generated random-numbers method	Allocation concealed from the participants and dietitians until randomization was revealed to the study participants at the initial intervention clinic appointment.	No	NR	5/44(4 for time limit/work schedule, 1 for rheumatic arthritis) in treatment group and 3/45(all for time limit/work schedule) in control group dropped out. Multiple imputations with the use of linear regression were used to impute missing values from 12 wk and were based on the assumption that data were missing at random.	None	No
Minami 2015	Computer-generated permuted-block randomisation	NR	Yes	NR	2/28 subjects in placebo group and 4/24 subjects in B-3 group dropped out for personal rea:	None	Authors describe no conflict of interest
Rabiei 2015	Stratified random sampling based on BMI	NR	Yes	Yes	40 of 46 participants completed the study, no reasons specified for dropping out.	None	PROTEXIN COMPANY and Nicootec company provided probiotic and placebo capsules
Reimer 2017	Computer-generated randomisation stratified accordng	An investigator not involved with subjects generated the randomization sequence	Yes	NR	3/31 in control (1 for time commitment, 2 did not respond to calls) and 3/31 in treatment g	None	Funded by General Mills Bell Insitute of Nutrition, a US based food company
Sanchez 2014	Computerised-generated randomisation system	NR	Yes	NR	28 subjects excluded after randomization, 32 subjects dropped out. Reasons for drop out we	None	Yes, sponsor (Nestle) did data analysis and data handling. Multiauthor paper with most authors from Nestle who prepared the maniuscript.
Sharafedtinov 2013	Computer generated 2: 1 randomization using SPSS	Blocked randomization lists produced by stati	Yes	NR	Data on 4 subjects in control group missing, reasons not specified	None	No, however corresponding author has patent to probiotic strain used in study
Stenman 2016	The randomization scheme(1:1:1:1 allocation) was generated using a computerized procedure into blocks of fourrandomizationcodes each.	Boxes of investigational product were labeled with the corresponding Randomization code and study centers were advised to always use the smallest available randomization code and corresponding study product. The randomization code was generated by the contract research organization.	Yes	Yes	Large number of dropout (91) but reasons specified well. Per protocol and intention to treat	None	Yes. This study was fully funded by DuPont Nutrition & Health. Each of the authors or their respective organizations were financially compensated by DuPont for their contribution in the study. In addition, M.S. is a shareholder of Clinical Research Services Turku, and M.C. and R.B.are founders and shareholders of Vaiomer.
Zarrati 2014	Computer generated blocked randomization stratified I	NR	Yes	NR	No dropouts as data is presented on all 75 subjects. No mention of issues with compliance.	None	Yogurt prepared by Dairy Industry Corporation. No conficts. No mention of sponsorship. Pegah) for preparation of the probiotic yogurt
