# Supplementary material for: Dietary Alteration of the Gut Microbiome and Its Impact on Weight and Fat Mass: A Systematic Review and Meta-Analysis
Source: Genes (Basel). 2018 Mar 16;9(3):167. doi: 10.3390/genes9030167 (PMC5867888; doi:10.3390/genes9030167)
Supplement: Supplementary file 1 [file genes-09-00167-s001.zip › Table S5.pdf]

Last Author	Journal	Year	Type of dietary agent	Agent	Duration	Placebo
Asemi	Annals of Nutrition and Metabolism	2013	Probiotic	Multispecies probiotic supplement consisted of 7 viable and freeze-dried strains: Lactobacillus acidophilus (2 × 10^9 CFU), L. casei (7 × 10^9 CFU), L. rhamnosus (1.5 × 10^9 CFU), L. bulgaricus (2 × 10^8 CFU), Bifidobacterium breve (2 × 10^{10} CFU), B. longum (7 × 10^9 CFU), Streptococcus thermophilus (1.5 ×10^9 CFU), and 100 mg fructo-oligosaccharide with lactose.	8 weeks	100 mg fructo-oligosaccharide with lactose.
Canfora	Gastroenterology	2017	Prebiotic	Galacto-oligosaccharide 15g	12 weeks	Maltodextrin 16.95g
Fernandes	Journal of Clinical Gastroenterology	2016	Prebiotic	Fructo-oligosaccharide 6g	15 days	Maltodextrin 6g
Fernandes	Journal of Clinical Gastroenterology	2016	Synbiotic	6 g of FOS+ 1x10^9 Lactobacillus paracasei LPC-37, 1 x 10^9 Lactobacillus rhamnosus HN001, 1x10^9 Lactobacillus acidophilus NCFM, and 1x10^9 Bifidobacterium lactis HN019	15 days	Maltodextrin 6g

Gomes	Obesity	2016	Probiotic	Maltodextrin (48.3%), modified starch (24.21%), xylitol (24.21%), silicium dioxide (0.97%), and $1x10^9$ CFU of each of the probiotic strains: Lactobacillus acidophilus LA-14, Lactobacillus casei LC-11, Lactococcus lactis LL-23, Bifidobacterium bifidum BB-06, and Bifidobacterium lactis BL-4 (DaniscoVR). The women consumed four sachets daily before breakfast for 8 weeks, totaling $2x10^{10}$ CFU/day.	8 weeks	Unspecified
Higashikawa	European Journal of Clinical Nutrition	2016	Probiotic	Living Pediococcus pentosaceus LP28 powder with dextrin, 10^{11}	12 weeks	Dextrin only
Higashikawa	European Journal of Clinical Nutrition	2016	Probiotic	Heat-killed Pediococcus pentosaceus LP28 powder with dextrin, 10^{11}	12 weeks	Dextrin only
Javadi	Crescent Journal of Medical and Biological Sciences	2017	Probiotic	Bifidobacterium longum and Lactobacillus acidophilus: $2x10^7$ CFU/d	3 months	Maltodextrin
Javadi	Crescent Journal of Medical and Biological Sciences	2017	Prebiotic	Inulin	3 months	Fat and lactose-free milk
Javadi	Crescent Journal of Medical and Biological Sciences	2017	Synbiotic	Bifidobacterium longum and Lactobacillus acidophilus: $2x10^7$ CFU/d + Inulin	3 months	Maltodextrin + Fat and lactose-free milk
Jung	Journal of Functional Foods	2015	Probiotic	2 g of powder of two probiotic strains, L. curvatus HY7601 and L. plantarum KY1032, each at 2.5×10^9 cfu, twice a day	12 weeks	2g powder (1.34 g of crystalline cellulose, 0.6 g of lactose, and 0.06 g of blueberry-flavoring agent)
Jung	Korean Journal of Family Medicine	2013	Probiotic	BNR17 capsules were composed of 10^{10} cfu of Lb. gasseri BNR17 and filler powder (50% trehalose, 25% skim milk, and 25% fructo-oligosaccharide) - 6 capsules/day	12 weeks	Filler alone

Kadooka	European Journal of Clinical Nutrition	2010	Probiotic	Lactobacillus gasseri SBT2055 $5x10^{10}$ cfu/100 g + 11% skim milk powder and a small amount of flavoring, agar and sucralose as a zero-calorie artificial sweetener	12 weeks	11% skim milk powder and a small amount of flavoring, agar and sucralose as a zero-calorie artificial sweetener
Kadooka	European Journal of Clinical Nutrition	2010	Probiotic	Lactobacillus gasseri SBT2055 $5x10^{10}$ cfu/100 g + 11% skim milk powder and a small amount of flavoring, agar and sucralose as a zero-calorie artificial sweetener	8 weeks	11% skim milk powder and a small amount of flavoring, agar and sucralose as a zero-calorie artificial sweetener
Kadooka	European Journal of Clinical Nutrition	2010	Probiotic	Lactobacillus gasseri SBT2055 $5x10^{10}$ cfu/100 g + 11% skim milk powder and a small amount of flavoring, agar and sucralose as a zero-calorie artificial sweetener	4 weeks	11% skim milk powder and a small amount of flavoring, agar and sucralose as a zero-calorie artificial sweetener
Kadooka	British Journal of Nutrition	2013	Probiotic	11% skimmed milk powder and a small amount of flavoring, agar and sucralose as a zero-energy artificial sweetener plus starter cultures of Streptococcus thermophilus and Lactobacillus delbrueckii ssp. Bulgaricus plus Lactobacillus gasseri SBT2055 7·9 (SD 1·1) x 10^{7}	12 weeks	11% skimmed milk powder and a small amount of flavoring, agar and sucralose as a zero-energy artificial sweetener plus starter cultures of Streptococcus thermophilus and Lactobacillus delbrueckii ssp. Bulgaricus
Kadooka	British Journal of Nutrition	2013	Probiotic	11% skimmed milk powder and a small amount of flavoring, agar and sucralose as a zero-energy artificial sweetener plus starter cultures of Streptococcus thermophilus and Lactobacillus delbrueckii ssp. Bulgaricus plus Lactobacillus gasseri SBT2055 6·9 (SD 3·5) x 106	12 weeks	11% skimmed milk powder and a small amount of flavoring, agar and sucralose as a zero-energy artificial sweetener plus starter cultures of Streptococcus thermophilus and Lactobacillus delbrueckii ssp. Bulgaricus

Kim	Food and Function	2017	Probiotic	2 g of probiotic powder twice a day containing L. curvatus HY7601 (2.5×10^9 colony forming units (cfu)) and L. plantarum KY1032 (2.5×10^9 cfu)	12 weeks	Powder without probiotic agents
Lambert	Clinical Nutrition	2017	Prebiotic	5 g/serving of yellow pea fiber ('Best' Pea Fiber, Best Cooking Pulses Inc., Portage la Prairie, MB, Canada)	12 weeks	isocaloric dose of control wafers with no pea fiber (204 kcal of total additional energy per day in each group).
Leber	European Journal of Clinical Nutrition	2012	Probiotic	Three bottles containing 65ml of YAKULT light (containing L. casei Shirota at a concentration of 10^8/ml, Yakult Austria, Vienna) per day for 3 months (probiotic)	3 months	None, no treatment
Madjd	American Journal of Clinical Nutrition	2016	Probiotic	PY was a commercially available product prepared with the starter cultures of Streptococcus thermophiles and Lactobacillus bulgaricus, enriched with the probiotic culture of 2 strains of lactobacilli (Lactobacillus acidophilus LA5) and bifidobacterial (Bifidobacterium lactis BB12) with a total minimum of $1X10^7$ colony-forming units.	12 weeks	nonprobiotic conventional yogurt contained the starter cultures of S. thermophiles and L. bulgaricus.
Minami	Journal of Nutritional Science	2015	Probiotic	Lyophilized powder of B. breve B-3, a strain originating from a healthy infant, and had mainly maize starch as the carrier in an acid-protective gelatin capsule (5×10^{10} colony-forming units per three capsules)	12 weeks	internal matrix (mainly maize starch).

Minami	Journal of Nutritional Science	2015	Probiotic	Lyophilized powder of B. breve B-3, a strain originating from a healthy infant, and had mainly maize starch as the carrier in an acid-protective gelatin capsule (5 × 10^{10} colony-forming units per three capsules)	8 weeks	internal matrix (mainly maize starch).
Minami	Journal of Nutritional Science	2015	Probiotic	Lyophilized powder of B. breve B-3, a strain originating from a healthy infant, and had mainly maize starch as the carrier in an acid-protective gelatin capsule (5 × 10^{10} colony-forming units per three capsules)	4 weeks	internal matrix (mainly maize starch).
Rabiei	Medical Journal of the Islamic Republic of Iran	2015	Synbiotic	Each symbiotic capsule was consisted of Lactobacillus casei, Lactobacillus rhamnosus, Streptococcus thermophilus, Bifidobacterium breve, Lactobacillus acidophilus, Bifidobacterium longum, Lactobacillus bulgaricus, FOS (Fructo-oligosaccharide - Prebiotic), Magnesium stearate (source: mineral and vegetable), Vegetable capsule (hydroxypropyl methyl cellulose) TVC: 200 million CFU TVC: 2 × 10^8 CFU.	12 weeks	Maltodextrin

Rabiei	Medical Journal of the Islamic Republic of Iran	2015	Synbiotic	Each symbiotic capsule was consisted of Lactobacillus casei, Lactobacillus rhamnosus, Streptococcus thermophilus, Bifidobacterium breve, Lactobacillus acidophilus, Bifidobacterium longum, Lactobacillus bulgaricus, FOS (Fructooligosaccharide - Prebiotic), Magnesium stearate (source: mineral and vegetable), Vegetable capsule (hydroxypropyl methyl cellulose) TVC: 200 million CFU TVC: 2 × 108 CFU.	6 weeks	Maltodextrin
Reimer	Molecular Research and Food Research	2017	Prebiotic	Prebiotic bar (inulin-type fructans (ITF) with 6 g oligofructose + 2 g inulin from chicory root);	12 weeks	Control bar
Sanchez	British Journal of Nutrition	2013	Probiotic	10 mg of a Lactobacillus rhamnosus CGMCC1.3724 powder providing 1·62 x 10^8 cfu, 300 mg of a mix of oligofructose and inulin (70:30, v/v) and 3mg of magnesium stearate, 2 capsules	12 weeks	250 mg of maltodextrin and 3mg of magnesium stearate
Sharafedtinov	Nutrition Journal	2013	Probiotic	L. plantarum TENSIA was added to the cheese milk in amounts of 1.5x10^{11} CFU/g before renneting.	3 weeks	Control cheese
Stenman	EBioMedicine	2016	Probiotic	Bifidobacterium animalis ssp. lactis 420, 10^{10} CFU/day in 12 g of microcrystalline cellulose (B420)	6 months	Placebo, microcrystalline cellulose 12 g/day;
Stenman	EBioMedicine	2016	Prebiotic	Litesse® Ultra polydextrose (LU) 12 g/day	6 months	Placebo, microcrystalline cellulose 12 g/day;
Stenman	EBioMedicine	2016	Synbiotic	Bifidobacterium animalis ssp. lactis 420, 10^{10} CFU/ day in 12 g of LU (LU + B420).	6 months	Placebo, microcrystalline cellulose 12 g/day;

Zarrati	Journal of the American College of Nutrition	2014	Probiotic	Probiotic yogurt was prepared with the starter cultures containing Streptococcus thermophilus and Lactobacillus bulgaricus and was enriched with the lactobacilli and bifidobacterial based probiotic cultures (Lactobacillus acidophilus LA5, Lactobacillus casei DN001, Bifidobacterium lactis BB12). The concentration of each probiotic strain in our yogurt was 1×10^7 colony-forming units/mL.	8 weeks	Conventional yogurt contained only starter cultures of S. thermophilus and L. bulgaricus
