# Supplementary figures and images for: Dietary Alteration of the Gut Microbiome and Its Impact on Weight and Fat Mass: A Systematic Review and Meta-Analysis
Source: Genes (Basel). 2018 Mar 16;9(3):167. doi: 10.3390/genes9030167 (PMC5867888; doi:10.3390/genes9030167)

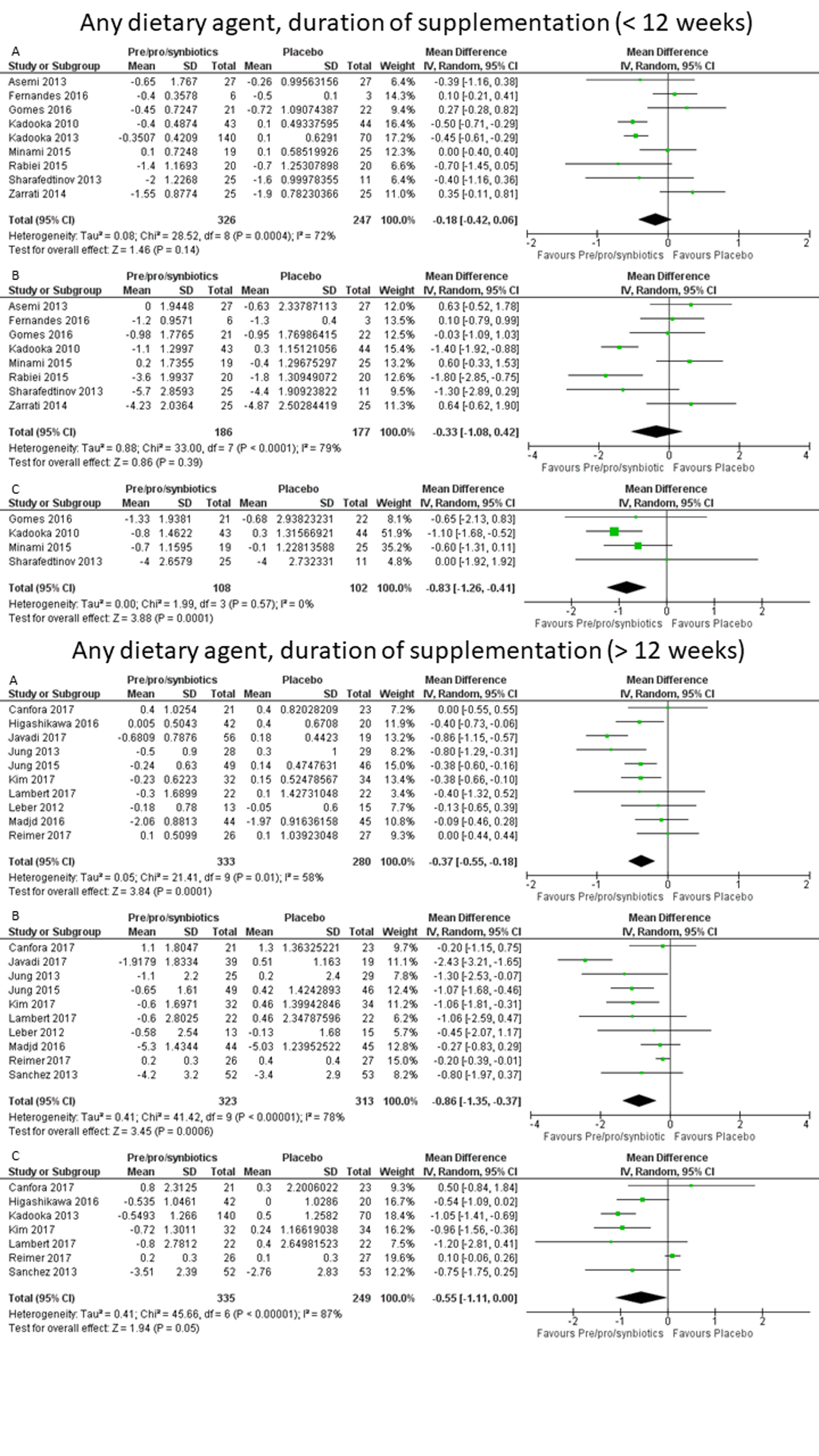

Supplement: Supplementary file 1 [file genes-09-00167-s001.zip › Fig S1.tif]

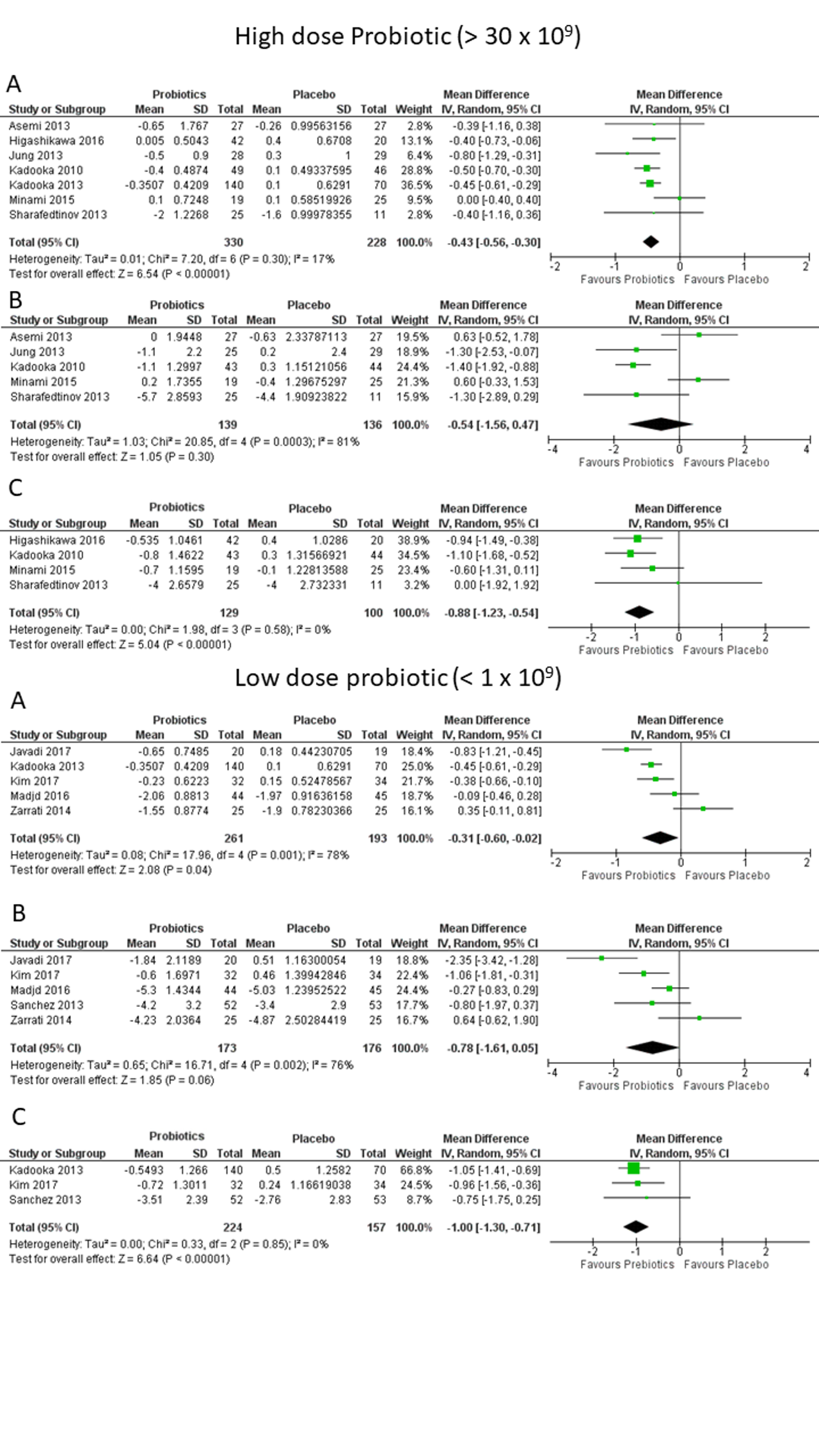

Supplement: Supplementary file 1 [file genes-09-00167-s001.zip › Fig S2.tif]

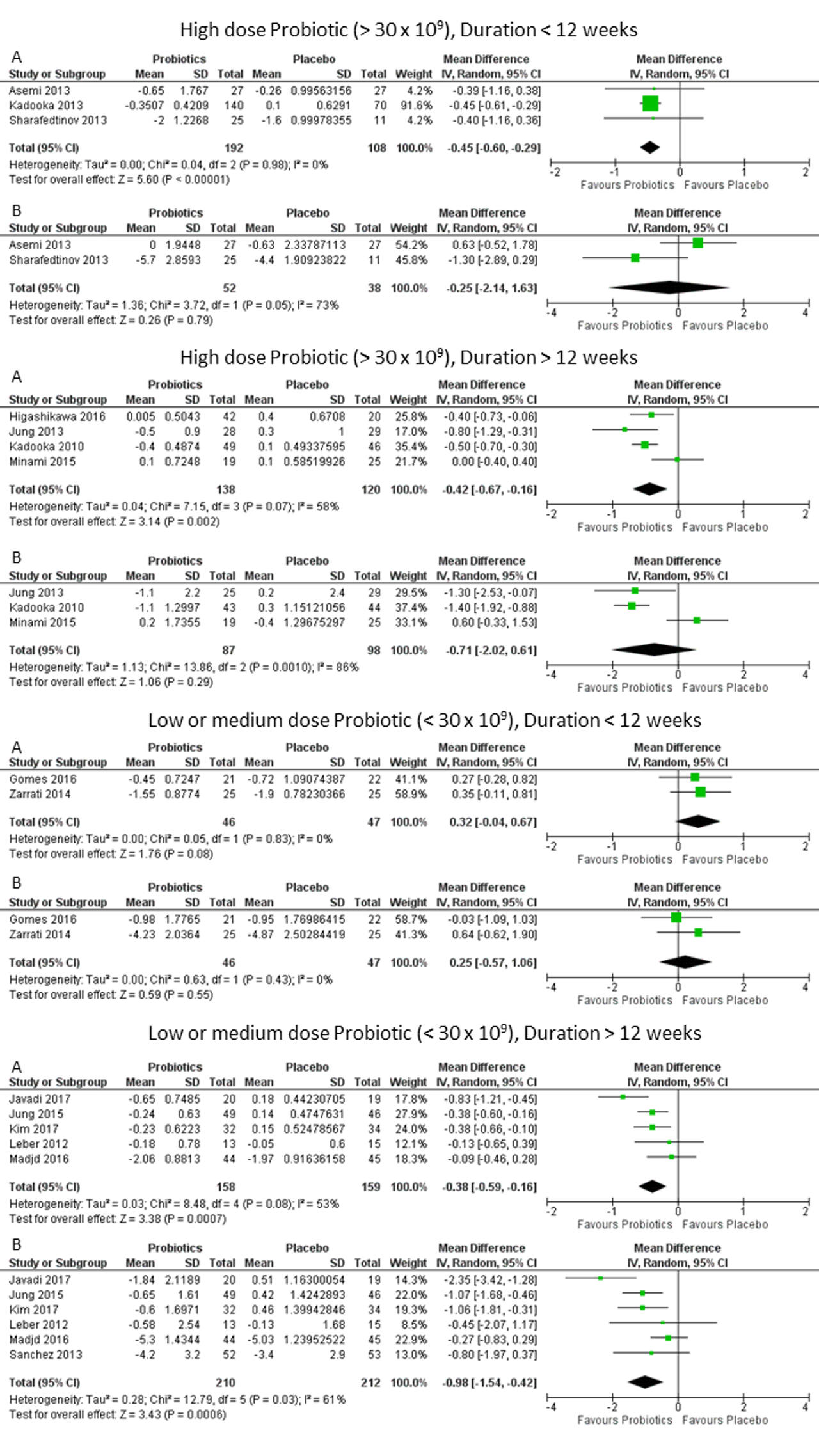

Supplement: Supplementary file 1 [file genes-09-00167-s001.zip › Fig S3.tif]

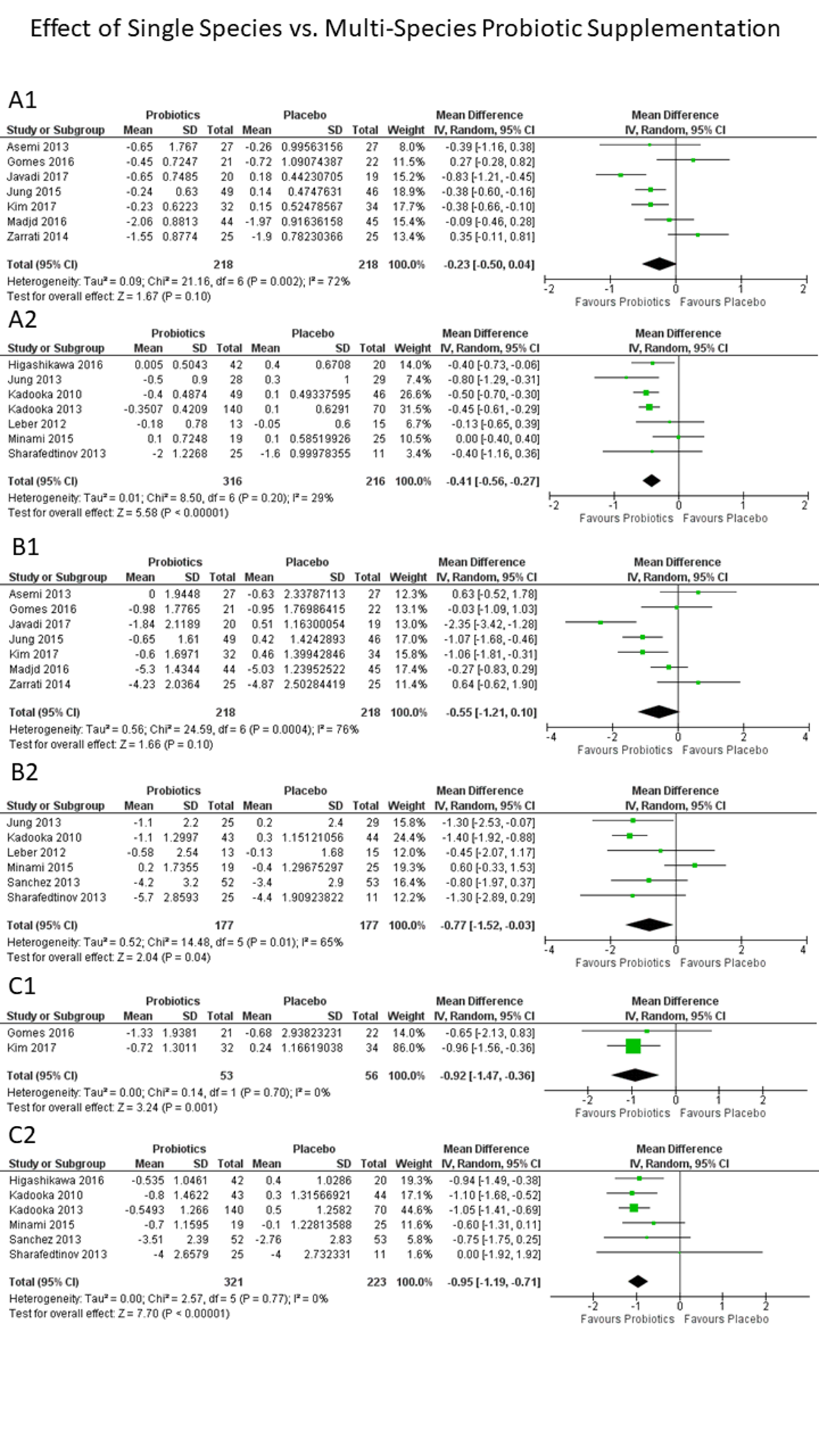

Supplement: Supplementary file 1 [file genes-09-00167-s001.zip › Fig S4.tif]

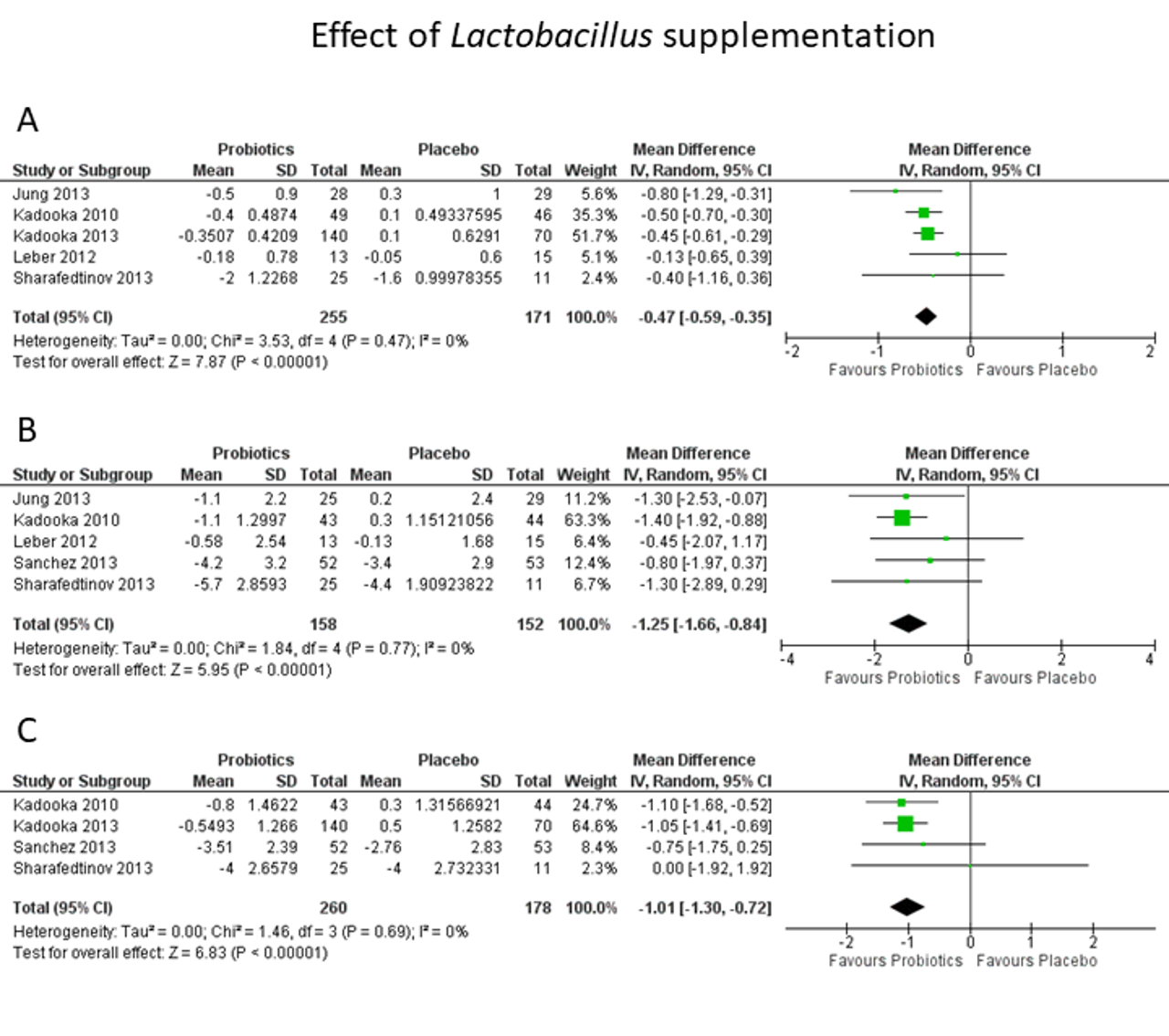

Supplement: Supplementary file 1 [file genes-09-00167-s001.zip › Fig S5.tif]

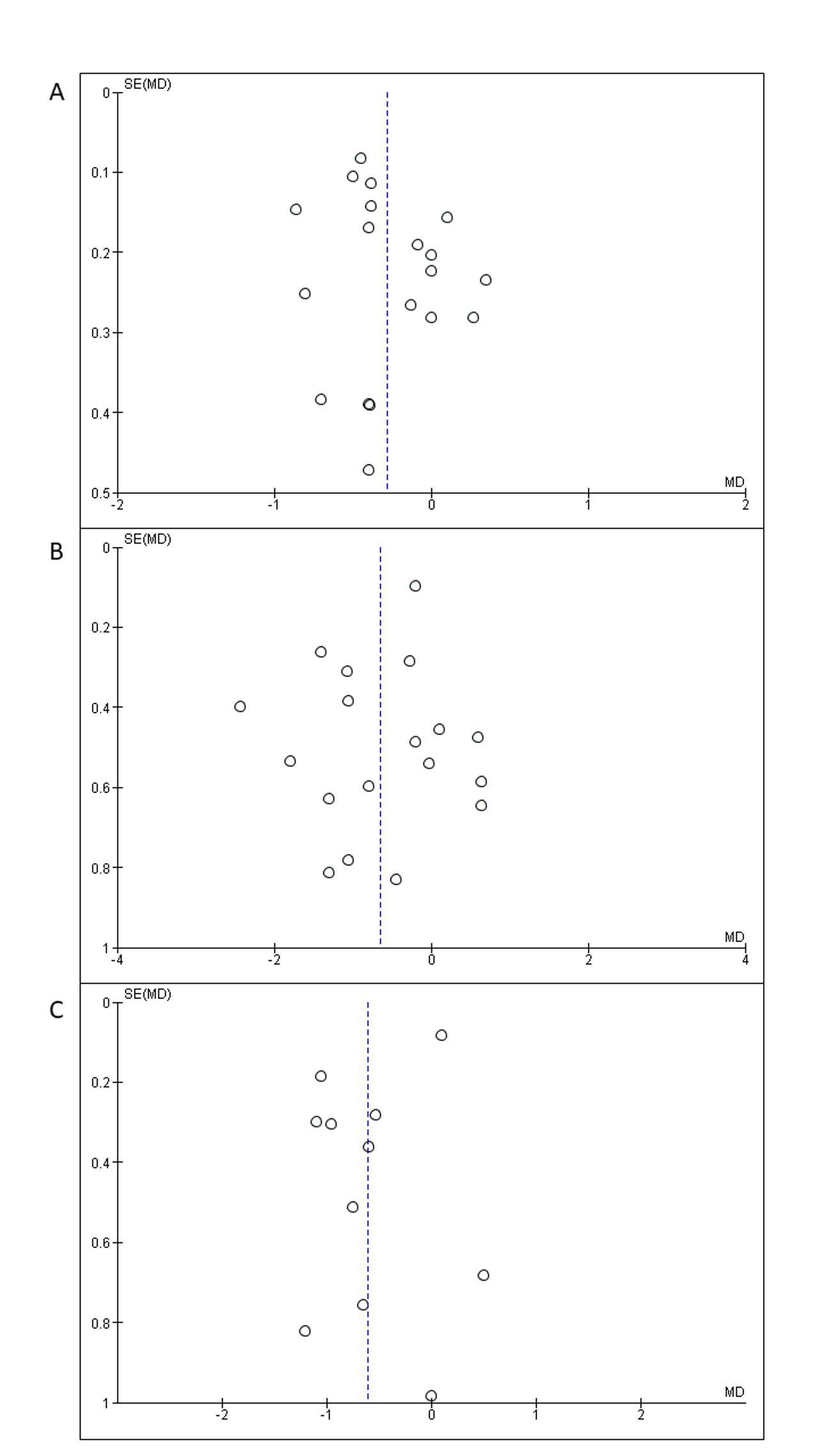

Supplement: Supplementary file 1 [file genes-09-00167-s001.zip › Figure S6.tif]

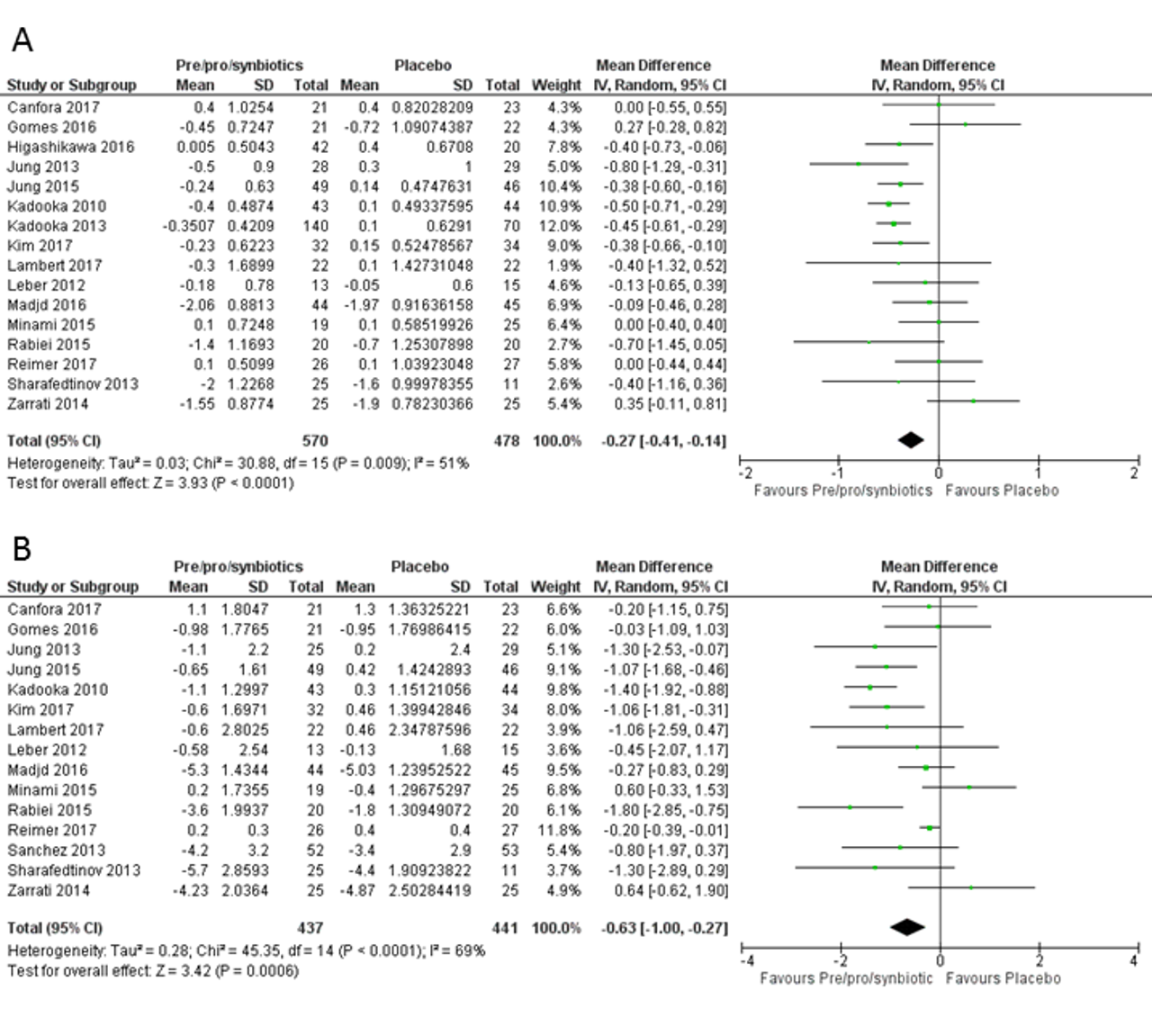

Supplement: Supplementary file 1 [file genes-09-00167-s001.zip › Figure S7.tif]
